# Development of a VHH that inhibits the binding of neuronal pentraxin 2 to a postsynaptic glutamate receptor, AMPAR

**DOI:** 10.1016/j.jbc.2025.110975

**Published:** 2025-11-25

**Authors:** Takanori Yokoo, Makoto Nakakido, Keiko Matsuda, Jose M.M. Caaveiro, Jorge Fernandez-Perez, Michisuke Yuzaki, Kouhei Tsumoto

**Affiliations:** 1 Department of Chemistry and Biotechnology, School of Engineering, The University of Tokyo, Bunkyo-ku, Tokyo, Japan; 2 Department of Bioengineering, School of Engineering, The University of Tokyo, Bunkyo-ku, Tokyo, Japan; 3 Department of Physiology, School of Medicine, Keio University, Shinjuku-ku, Tokyo, Japan; 4 Department of Protein Drug Discovery, Graduate School of Pharmaceutical Sciences, Kyushu University, Higashi-ku, Fukuoka, Japan; 5 The institute of Medical Science, The University of Tokyo, Minato-ku, Tokyo, Japan

**Keywords:** neuronal pentraxin, ESPs, synaptic organizer, AMPAR, nanobody, mass photometry, X-ray crystallography, chronic itch, Alzheimer's disease

## Abstract

Neurons connect to each other *via* synapses to form neural circuits. Recent research has shown that neuropsychiatric disorders and neurological disorders, such as autism spectrum disorders and Alzheimer's disease, are synaptic diseases caused by abnormalities of synapses. Synaptic organizers are molecules responsible for synapse formation. Neuronal pentraxin 2 (NP2) is a synaptic organizer and a secreted protein that is expressed mainly in the hippocampus and cerebellum, and it contributes to synaptic plasticity. NP2 forms clusters with its family proteins, NP1 and neuronal pentraxin receptor, and binds to postsynaptic amino-3-hydroxy-5-methyl-4-isoxazolepropionic acid–type receptors. In recent years, research has revealed the disease relevance of NP2. For example, it can be a biomarker of Alzheimer's disease, and its overexpression in the peripheral nervous system has been reported to cause chronic itch. However, the mechanism of NP2 function has not been well described at the molecular level. In this study, we developed a variable domain of a heavy-chain antibody (VHH) against NP2 to elucidate its molecular mechanism of action and to regulate its function of NP2. The obtained VHH N1 showed high specificity and affinity to NP2, and its binding mechanism was elucidated by X-ray crystallography. Furthermore, VHH N1 inhibited the binding of NP2 to amino-3-hydroxy-5-methyl-4-isoxazolepropionic acid–type receptors, and this inhibitory activity was confirmed in cells. These results provide useful insights into the molecular mechanism of NP2 function and highlight the potential application of VHH N1 as a detection agent for NP2 or as a therapeutic agent for chronic itch.

Neurons connect to each other *via* synapses to form neural circuits, and the human brain is believed to have about 10^14^ synaptic connections formed between about 10^11^ neurons (1). Neuropsychiatric disorders and neurological disorders, such as autism spectrum disorders, epilepsy, schizophrenia, and Alzheimer's disease (AD), are considered to be synaptic diseases caused by an imbalance between excitatory and inhibitory synaptic function (2, 3, 4, 5, 6). The formation and maintenance of neural circuits is carried out by a group of proteins called synaptic organizers (7, 8). Cell adhesion molecules, such as neurexins, neuroligins (9, 10), and MAM domain–containing glycosylphosphatidylinositol anchor protein (MDGA1), as well as diffusion factors, such as fibroblast growth factor (11, 12) and Wnt (13, 14), which are secreted and diffuse to work, are molecules responsible for developmental processes. Recently, a group of molecules called extracellular scaffold proteins (ESPs), which directly link presynaptic and postsynaptic membrane proteins, have been identified as proteins responsible for synapse formation and maintenance in the mature brain (7).

Neuronal pentraxins (NPs) are ESPs that bind to amino-3-hydroxy-5-methyl-4-isoxazolepropionic acid–type receptors (AMPARs), which consist of GluA1–4 subunits (7), and mediate excitatory neurotransmission in the central nervous system (15). NPs also play a major role in synaptic plasticity in the cerebellum and the hippocampus. The NP family consists of Nptx1 (NP1), Nptx2 (NP2), and Nptxr (neuronal pentraxin receptor [NPR]) and belongs to a pentraxin family that originated early in vertebrate divergence (16). In mice, NP1 (17) and NPR (18) mRNAs are highly expressed in the hippocampus (CA3 and dentate gyrus), cerebellum (Purkinje cells and granule cells), and cortex. NP2 mRNA expression occurs in similar brain regions (19, 20). NPs have been shown to cluster AMPARs *via* pentraxin domains (PTXs) (20, 21, 22). Indeed, overexpression of exogenous NPs induced clustering of postsynaptic AMPARs *in vitro* (20, 21, 23), indicating that Nptxs function as ESP-type postsynaptic organizers.

NP1 and NP2 are secreted proteins, whereas NPR is a type II transmembrane protein. The full-length NP1 and NP2 and the extracellular region of NPR consist of coiled-coil 1 and 2 (CC1 and CC2) and a PTX from the N terminus to the C terminus (21). Only the PTX domain of NP1 has been structurally characterized (24). NPs are thought to form homomeric and heteromeric complexes *via* disulfide bonds using three cysteines in CC1 (21), and they have been reported to exist as pentamers (25) or hexamers (21). The first and second cysteines from the N terminus have been reported to form disulfide bonds in hexamers, and the third cysteine has been thought to form disulfide bonds among hexamers (21). NP1 and NP2 are likely anchored on the membrane *via* oligomerization with the membrane protein NPR (7, 21). NP oligomers anchored on the membrane *via* NPR are excised from the membrane and secreted *via* controlled cleavage by the matrix metalloproteinase tumor necrosis factor alpha–converting enzyme, and this soluble NP oligomer binds to the amino-terminal domain (ATD) of GluAs (20). In particular, Suzuki et al. (24) reported that NPs bind to the GluA4 subunit with micromolar order affinity.

Recently, researchers have described the relationship between the expression level of NP2 and diseases. For example, NP2 was found to be downregulated in AD patients, and it may prove to be a marker gene for AD (26, 27). Furthermore, NP2 is also expressed in peripheral nerves, and *in vivo* experiments showed that overexpression of NP2 in peripheral nerves causes chronic itch (28, 29). Despite the importance of NP2, its function has not yet been elucidated at the structural level or the molecular level. Clarification of the function of NP2 would lead to a better understanding of synaptogenesis and could potentially lead to the generation of novel therapeutics.

Variable domain of a heavy-chain antibody (VHH) (*i*.*e*., a nanobody) is a single-domain antibody extracted from the variable regions of the heavy-chain antibody derived from camelids (30). VHHs retain high affinity and specificity toward various antigens despite the single-domain architecture. Due to their small molecular size (30), VHHs can be used to investigate the functions of NP2 even in the narrow space of the synaptic cleft, which has a width of ∼20 nm (31).

In this study, we generated a VHH that binds specifically to NP2 with affinities on the order of nanomolar. We determined the crystal structure of the complex of NP2 PTX and VHH N1 and described the molecular recognition of NP2 PTX by VHH N1. We also assessed the effect of VHH N1 on the binding of NP2 to AMPARs and the oligomerization of NP2. Based on our results, we discuss the molecular mechanism of NP2 function.

## Results

### Characterization of NP2

**Figure 1:**
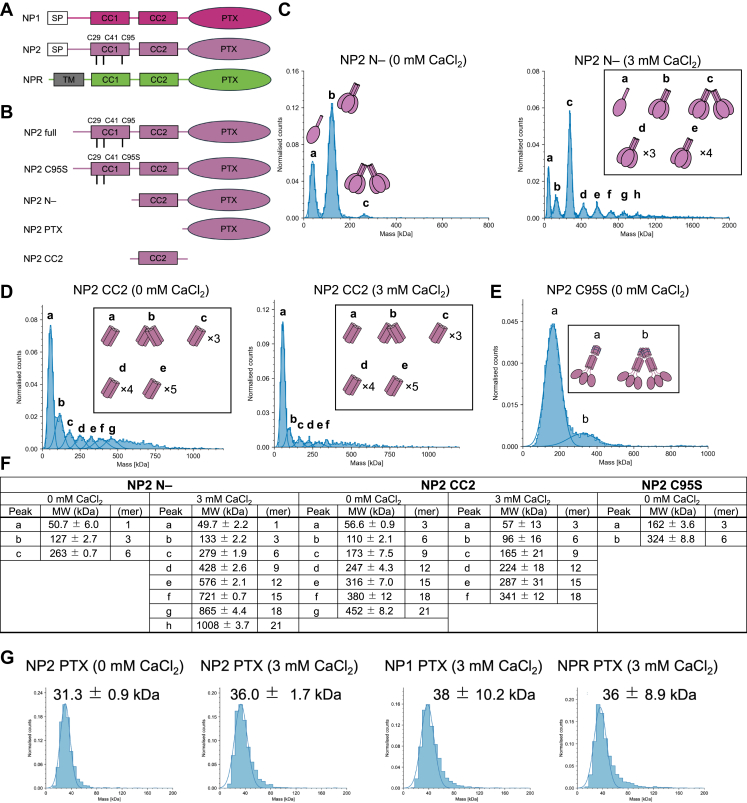
**Constructs and oligomerization state of NPs**. *A*, domain structure of NP1, NP2, and NPR. *B*, constructs of NP2 used in this study. *C* and *D*, frequency distribution of molecular weight of (*C*) NP2 N- and (*D*) NP2 CC2 in the presence or the absence of Ca^2+^ detected by mass photometry. *E*, frequency distribution of molecular weight of NP2 C95S. *F*, summary of the determined molecular weight and oligomerization state of NP2 N-, NP2 CC2, and NP2 C95S. *G*, frequency distribution of molecular weight of NP1 PTX, NP2 PTX, and NPR PTX detected by mass photometry. Measurements were conducted using a buffer composed of 10 mM Hepes–NaOH (pH 7.4) and 150 mM NaCl with or without 3 mM CaCl_2_ at 25 °C. Each measurement was performed three times independently. NP, neuronal pentraxin; NPR, neuronal pentraxin receptor; PTX, pentraxin domain.

NP2 consists of CC1 and CC2 at the N terminus and a PTX domain at the C terminus (Fig. 1*A*). We attempted to prepare the full-length Nptx2 as a recombinant protein using a mammalian expression system but failed to obtain purified protein because of extremely low secretion to culture medium and aggregation during purification steps. We hypothesized that CC1, which contributes significantly to the oligomerization of NP2 and forms several disulfide bonds using cysteines (C30, C42, and C95 numbering includes signal peptide), may result in the aggregation of NP2, and prepared NP2 N-, in which CC1 was deleted. We also prepared the PTX domain of NP1, NP2, and NPR (Fig. 1*B*). Each construct was successfully expressed and purified using the mammalian expression system. Size-exclusion chromatography (SEC) profiles indicated that the PTX domain alone exists as a monomer, whereas NP2 N- forms oligomers (Fig. S2).

We evaluated the oligomerization state of NP2 N- by mass photometry (MP) and found that it is in equilibrium between hexamer, trimer, and monomer, and the proportion of trimer is highest among them (Fig. 1*B*). Because the crystal structure of NP1 PTX (Protein Data Bank [PDB] ID: 6YPE) showed that the PTX domain has two calcium ion (Ca^2+^)–binding sites (24), we assessed whether the molecular state of NP2 N- changes in the presence of Ca^2+^. The results revealed the simultaneous presence of different highly oligomerized states, which were not observed in the absence of Ca^2+^ (Fig. 1, *C* and *F*). The calculation of molecular weight corresponding to each peak showed that the molecular weight increased regularly by approximately 140 kDa for peaks that were larger than the monomers and trimers. This result indicated that trimers are the smallest units that form various oligomerization states, such as hexamers, nonamers, and dodecamers (Fig. 1*F*). In contrast, MP analysis indicated that NP2 PTX exists as a monomer regardless of the presence of Ca^2+^ (Fig. 1*G*), which suggests that the high degree of oligomerization of NP2 N- is driven by the N-terminal CC regions.

To evaluate the contribution of the CC2 region, we prepared the construct consisting of only the CC2 region (NP2 CC2) and assessed the clustering behavior in the presence or the absence of Ca^2+^. NP2 CC2 formed various oligomers with the trimer as the smallest unit, like NP2 N-. Notably, this oligomerization occurred in a Ca-independent manner, suggesting that the PTX domain would have a role in controlling the Ca^2+^-dependent oligomerization of NP2.

Furthermore, to prepare full-length recombinant NP2, we designed a single mutant where cysteine 95 in CC1, which is thought to contribute to the formation of disulfide bonds between hexamers, was mutated to serine (C95S). Although the yield was still very low, we managed to successfully purify the protein of NP2 C95S and investigated the oligomerization of the construct by MP analyses.

In the absence of CaCl_2_, the result of MP analyses suggested that NP2 C95S exists as a trimer or a hexamer (Fig. 1*E*). Compared with NP2 N-, which lacks CC1, the absence of monomer suggests that the presence of CC1 made the oligomers more stable and robust, which is consistent with a previous study (21). On the other hand, the addition of Ca^2+^ caused rapid aggregation and precipitation, and the sample was not able to be subjected to MP analysis. Taken together with that NP2 C95S exists as a trimer or a hexamer in the absence of Ca^2+^ as described above, the result indicates that NP2 C95S, like NP2 N-, aggregates with the trimer as its smallest unit in the presence of Ca^2+^, but the oligomerization rate is significantly faster, which is also consistent with previous research highlighting the importance of C95 on the oligomerization of NP protein (21).

### Generation of VHHs against NP2

We generated VHHs against NP2 as a tool for functional analyses and to regulate the function of NP2. To obtain VHHs that bind to various regions of NP2, considering the yield of each construct, an alpaca was immunized with NP2 N-. We constructed an immune library and isolated VHH N1 as the candidate binder against NP2 by panning *via* phage display from the library (Fig. S3). We expressed and purified VHH N1 as a recombinant protein and evaluated its interaction with NP2 using surface plasmon resonance (SPR) and isothermal calorimetry (ITC).

**Figure 2:**
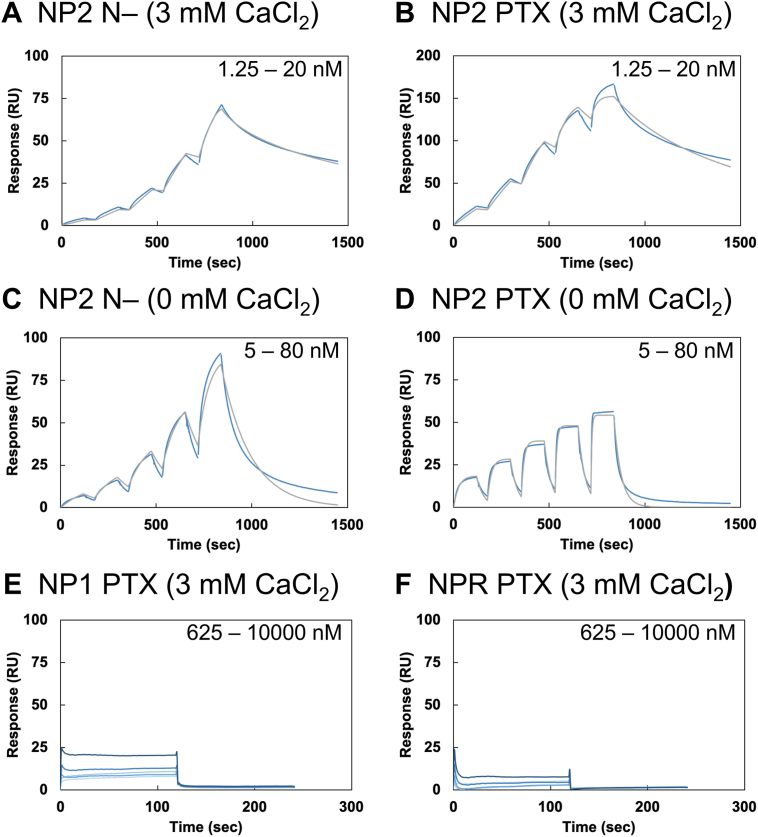
**SPR sensorgrams of the interaction between VHH N1 and NP constructs**. *A*–*D*, the SPR sensorgrams of the interaction between NP2 constructs and VHH N1. *A*, against NP2 N- in the presence of CaCl_2_. *B*, against NP2 PTX in the presence of CaCl_2_. *C*, against NP2 N- in the absence of CaCl_2_. *D*, against NP2 PTX in the absence of CaCl_2_. The measurements were conducted by the single-cycle kinetics method. The *blue line* shows raw responses, and the *gray line* shows the fitting curves. *E* and *F*, the SPR sensorgrams of the interaction between NP1 or NPR PTX and VHH N1. *E*, against NP1 PTX in the presence of CaCl_2_. *F*, against NPR PTX in the presence of CaCl_2_. The measurements were conducted using the multicycle kinetics method. The *blue lines* show the raw responses with different concentrations. All SPR measurements were performed in 10 mM Hepes–NaOH at pH 7.4, 150 mM NaCl, and Tween-20 (0.005%) with or without 3 mM CaCl_2_ at 25 °C. NPs were immobilized on a CM5 sensor chip, and VHH N1 was flowed as the analyte. Each measurement was performed three times independently, and each figure shows a representative sensorgram of triplicated experiments. NP, neuronal pentraxin; NPR, neuronal pentraxin receptor; PTX, pentraxin domain; SPR, surface plasmon resonance; VHH, variable domain of a heavy-chain antibody.

SPR results showed that VHH N1 bound to NP2 N- with high affinity (*K*_D_ = 1.41 nM, Figure 2*A*, Table 1), and similar binding was observed against NP2 PTX (Fig. 2*B*). These results indicate that the PTX domain is the epitope of VHH N1. In the absence of Ca^2+^, affinity was several 10 times lower against both NP2 N- and NP2 PTX (Fig. 2, *C* and *D*), and the dissociation rate against NP2 PTX increased. Crossreactivity was further evaluated by SPR measurements using NP1 PTX and NPR PTX, whose sequence homology with NP2 PTX is approximately 70%. The bindings of VHH N1 to NP1 PTX (Fig. 2*E*) and NPR PTX (Fig. 2*F*) were negligible, demonstrating the high specificity of the antibody against NP2.

**Table 1:** Kinetic parameters of VHH N1 evaluated by SPR

NP constructs	*k*_on_ (10^5^/Ms)	*k*_off_ (10^-2^/s)	*K*_*D*_ (nM)
NP2 N-1 (+Ca^2+^)	37.0 ± 0.8	0.524 ± 0.27	1.41 ± 0.05
NP2 N- (–Ca^2+^)	1.56 ± 0.07	0.514 ± 0.069	33.5 ± 6.1
NP2 PTX (+Ca^2+^)	19.2 ± 0.2	0.208 ± 0.018	1.14 ± 0.25
NP2 PTX (–Ca^2+^)	22.1 ± 0.3	2.49 ± 0.07	11.6 ± 0.3
NP1 PTX (+Ca^2+^)	ND	ND	ND
NPR PTX (+Ca^2+^)	ND	ND	ND

All data are the average of three independent SPR analyses, respectively.

ND, not determined.

**Figure 3:**
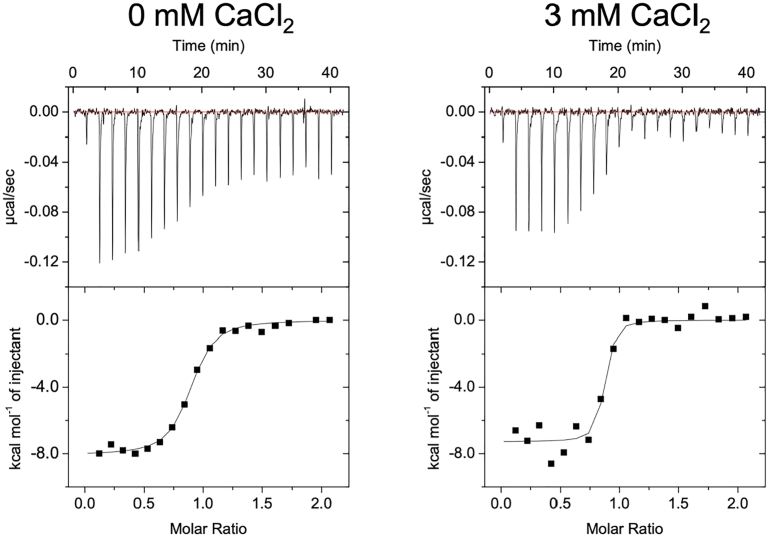
**ITC titration curves of the interaction between VHH N1 and NP2 PTX**. *Left*, in the absence of CaCl_2_. *Right*, in the presence of CaCl_2_. ITC measurements were conducted in 10 mM Hepes–NaOH (pH 7.4) and 150 mM NaCl with or without 3 mM CaCl_2_ at 25 °C. VHH N1 was added to the cell, and NP2 PTX was injected from the syringe. Each titration consisted of 20 injections. Each measurement was performed three times independently. ITC, isothermal calorimetry; NP, neuronal pentraxin; PTX, pentraxin domain; VHH, variable domain of a heavy-chain antibody.

We also conducted ITC analysis to further characterize the interaction (Fig. 3, Table 2). Because both the calculated enthalpy change value (ΔH) and entropy change value (ΔS) were energetically favorable, it was suggested that the interaction was driven by both enthalpy and entropy. The affinity values calculated by SPR are higher than the ITC values (*K*_D__ITC_/*K*_D__SPR_ of NP2 PTX in the presence of Ca^2+^ was 17.3), probably because of the difference in the molecular state of NP2 due to immobilization in SPR analyses.

**Table 2:** Thermodynamic parameters of VHH N1 evaluated by ITC

Concentration of CaCl_2_	Binding ratio (N)	*K*_D_ (nM)	ΔG (kcal/mol)	ΔH (kcal/mol)	–TΔS (kcal/mol)
0 mM CaCl_2_	0.781 ± 0.044	57.5 ± 6.5	−9.88 ± 0.06	−7.53 ± 0.31	−2.36 ± 0.34
3 mM CaCl_2_	0.913 ± 0.045	19.7 ± 9.0	−10.62 ± 0.26	−7.36 ± 0.24	−3.28 ± 0.48

All data are the average of three independent ITC analyses, respectively.

Moreover, affinity was lower in the absence of Ca^2+^, which is consistent with SPR results. The enthalpy gain did not change, but the entropy gain increased in the presence of Ca^2+^, suggesting that the binding mode of VHH N1 did not change regardless of the presence of Ca^2+^. It is possible that the Ca^2+^ binding would restrict the conformational change of unbound NP2 PTX to become more rigid and thereby decrease unfavorable entropy loss upon the VHH N1 binding.

Together, the SPR and ITC results showed that VHH N1 has both high affinity and specificity toward NP2 PTX.

### Cell imaging using VHH N1

**Figure 4:**
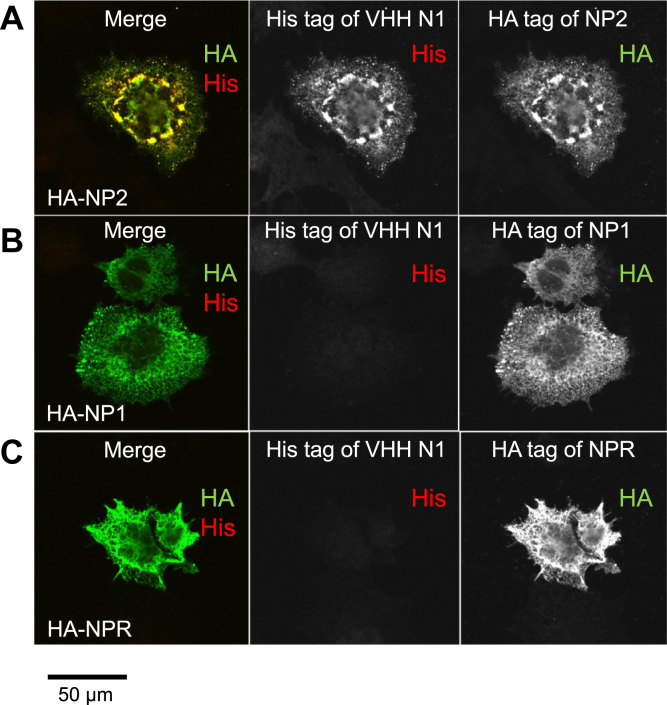
**VHH N1 recognized NP2 expressed in HEK cells with high sensitivity and high specificity**. Full-length NP2 (*A*), NP1 (*B*), or extracellular domain of NPR (*C*) with HA-tag expressed in HEK293 cells were stained with mouse anti-HA (*green*) to confirm expression and His-tagged VHH N1 followed by rabbit anti-His staining (*red*), showing specific binding of VHH N1 on HA-NP2. The scale bar represents 50 μm. HA, hemagglutinin; HEK293, human embryonic kidney 293 cell line; NP, neuronal pentraxin; NPR, neuronal pentraxin receptor; VHH, variable domain of a heavy-chain antibody.

To assess the specificity of VHH N1 for NP2, we overexpressed full-length NP1, NP2, or the extracellular domain of NPR, each tagged with hemagglutinin (HA), in human embryonic kidney 293 (HEK293) cells. The cells were stained with 1 μg/ml of VHH N1 and detected *via* a His-tag. HA-tag staining was used as a control to confirm the expression of each NP. VHH N1 signal was observed exclusively in cells expressing NP2, indicating that VHH N1 specifically recognizes NP2 in cells (Fig. 4, *A*–*C*).

### Crystal structure of VHH N1 and NP2 PTX

**Figure 5:**
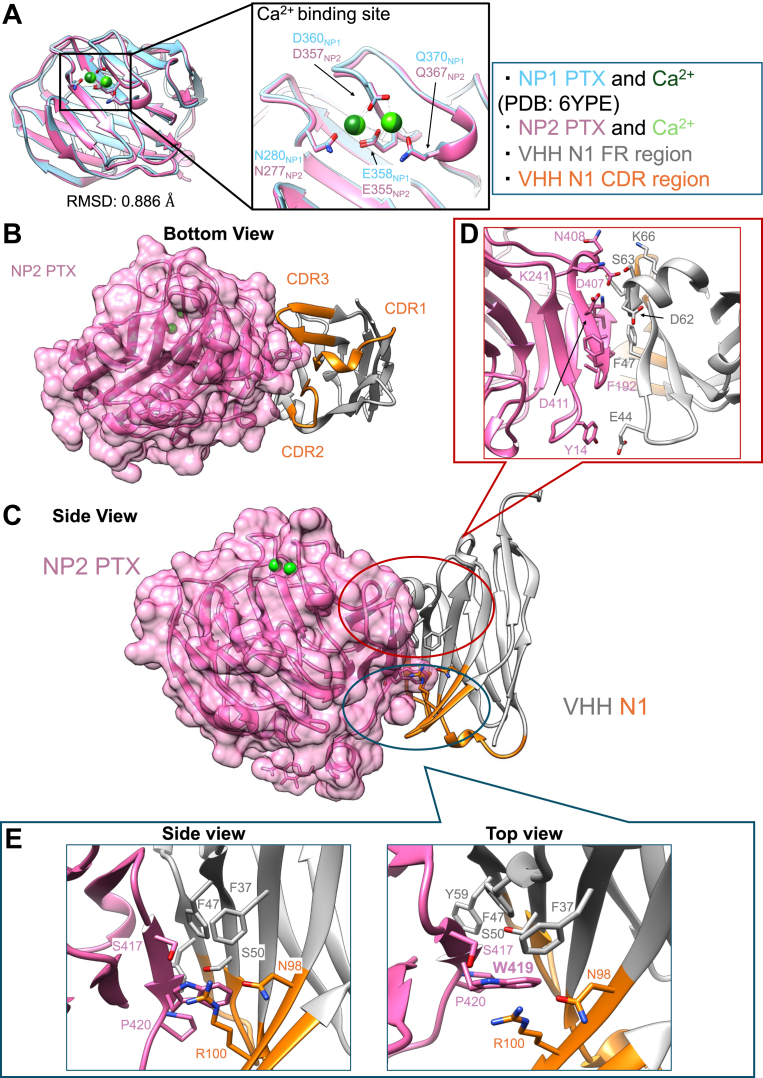
**Crystal structure of NP2 PTX in complex with VHH N1**. *A*, comparison of the NP2 PTX structure with NP1 PTX (Protein Data Bank ID: 6YPE). NP1 PTX is shown in *light blue*, and NP2 PTX is shown in *pink*. (*B*) *Bottom* view and (*C*) side view of the complex structure of NP2 PTX and VHH N1. The CDR regions of VHH N1 are depicted in *orange*, and the framework region (FR) of VHH N1 is shown in *gray*. *D*, interaction interface between NP2 PTX and the FR. *E*, core interaction interface around W419_NP2_. VHH N1 strongly surrounded W419_NP2_ using its FR2 and CDR3 region. CDR, complementarity-determining region; NP, neuronal pentraxin; PTX, pentraxin; VHH, variable domain of a heavy-chain antibody.

To identify the epitope of VHH N1, we used X-ray crystallography to determine the high-resolution complex structure of VHH N1 and NP2 PTX (Fig. 5). We then compared the structure of NP2 PTX with the previously reported NP1 PTX structure (PDB ID: 6YPE) (Fig. 5*A*) (24). The overall structure of NP2 PTX was quite similar to that of NP1 PTX (the RMSD value was 0.886 Å). Even though purification and crystallization of NP2 PTX and VHH N1 were conducted using a buffer lacking Ca^2+^, two Ca^2+^ ions were observed in the NP2 PTX domain, and the positions corresponded to the Ca^2+^ binding sites found in the crystal structure of NP1 PTX. VHH N1 bound to the side of NP2 PTX away from the two Ca^2+^ binding sites (Fig. 5, *B* and *C*). However, considering that the affinity of VHH N1 changed in the presence of Ca^2+^, there might be another, third Ca^2+^ binding site in the PTX domain of NP2. Taken together with that affinity improvement was from entropy gain, the Ca^2+^ binding site would not be located at the interaction interface and rather affect the dynamics of the PTX domain. The epitope region of VHH N1 straddles the N and C termini of the PTX domain. The molecular recognition of NP2 PTX is very unique in that the contribution of the complementarity-determining region 3 (CDR3) is very small (buried surface area_CDR3_ <100 *Å*^2^) compared with that of typical VHHs (Tables S1 and S2), in which CDR3 plays an important role in antigen recognition (32), and CDR2, framework region 2 (FR2), and FR3 regions tuck the β-sheet region of NP2 PTX (Fig. 5*D*). There are no salt bridge formations and only a few hydrogen bonds between VHH N1 and NP2 PTX. This result explains the relatively small heat generation observed in ITC analysis.

Amino acid residues in CDR3 and the FR2 region of VHH N1 surrounded a tryptophan residue W419_NP2_ (Fig. 5*E*), forming a core interface between VHH N1 and NP2 PTX. In NPR, a tryptophan residue corresponding to W419_NP2 PTX_ is not conserved, suggesting that recognition of this tryptophan residue would be critical for specificity against NPR. On the other hand, in NP1 PTX, two residues forming a hydrogen bond with VHH N1 (D407_NP2 PTX_ and V412_NP2 PTX_) are replaced with glutamic acid and isoleucine, respectively. To validate the contribution of these residues to the interaction with VHH N1, we prepared the single alanine mutants (D407A, V412A, and W419A) of NP2 PTX and conducted the SPR analyses (Fig. S5 and Table S3). In D407A, affinity was three times lower than that of NP2 PTX WT, suggesting the loss of hydrogen bonds with VHH N1. In V412A_NP2_, affinity got several 10 times lower than that of NP2 PTX WT because of the significantly faster dissociation (Fig. S5 and Table S3). Considering that the expression level and the yield of NP2 PTX V412A_NP2_ considerably dropped compared with NP2 PTX WT, V412_NP2_ would have roles not only in forming hydrogen bond with VHH N1 but also in stabilizing the structure of NP2 PTX (Fig. S5 and Table S3). In W419A_NP2_, binding got significantly lower, and we could not determine the affinity and kinetic parameters, validating the importance of this core interaction (Fig. S5). Taken together with the interaction analyses, the recognition of these residues is critical to generate the specificity of VHH N1 against NP2.

### Inhibitory activity of VHH N1 against NP2 binding to GluA

**Figure 6:**
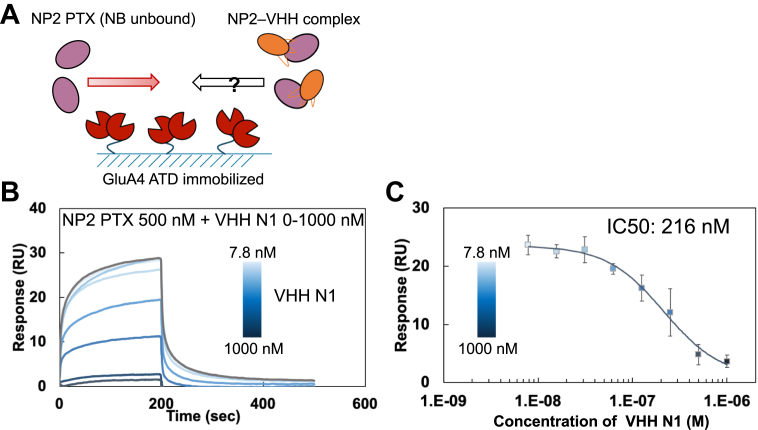
**VHH N1 inhibited the binding of NP2 to GluA4 ATD**. *A*, overview of the assay. The GluA4 ATD was immobilized on the SPR sensor chip, and samples of 500 nM NP2 PTX mixed with 0 nM to 1000 nM of VHH N1 were injected as analytes. *B*, SPR sensorgram of the interaction between GluA4 ATD and NP2 PTX in the presence of VHH N1. The sensorgram of NP2 PTX without VHH N1 is colored *gray*. *C*, relationship between the binding response of NP2 PTX against GluA4 and the concentration of VHH N1. SPR measurements were performed in 10 mM Hepes–NaOH at pH 7.4, 150 mM NaCl, and Tween-20 (0.005%) with or without 3 mM CaCl_2_ at 25 °C. Runs were performed using the single kinetics method. The measurement was performed four times independently. ATD, amino-terminal domain; NP, neuronal penntraxin; PTX, pentraxin; SPR, surface plasmon resonance; VHH, variable domain of a heavy-chain antibody.

Members of the NP family reportedly play an important role in synaptogenesis by interacting with AMPARs GluA1–4, especially GluA4, *via* the PTX domain (24, 33, 34). We first validated the interaction between NP2 and GluA4, and the determined affinity of NP2 PTX against GluA4 was 13.6 μM, which is similar to the reported affinity of NP1 PTX against GluA4 (Fig. S5) (24). Furthermore, NP2 N- did not bind to GluA4 ATD in the absence of Ca^2+^. On the contrary, in the presence of Ca^2+^, NP2 N- bound to GluA4 ATD, although kinetic parameters could not be calculated because of the mixture of different association states of NP2, as shown in Figure 1. In addition, we revealed that NP2 CC2 showed no binding to GluA4, suggesting that at least CC2 is not involved in the binding, and that the slower dissociation rate in NP2 N- compared with NP2 PTX was due to the avidity effect of multimerization. Subsequently, we assessed the ability of VHH N1 to inhibit the interaction between NP2 and GluA4 using SPR. The GluA4 ATD was immobilized on a sensor chip, and a constant concentration of NP2 PTX and various concentrations of VHH N1 were added simultaneously as analytes. We found that the binding response decreased in a VHH N1 concentration–dependent manner (Fig. 6), indicating that VHH N1 inhibited the binding of NP2 to GluA4 (IC_50_ = 216 nM).

### Inhibitory activity of VHH N1 against NP2 oligomerization

**Figure 7:**
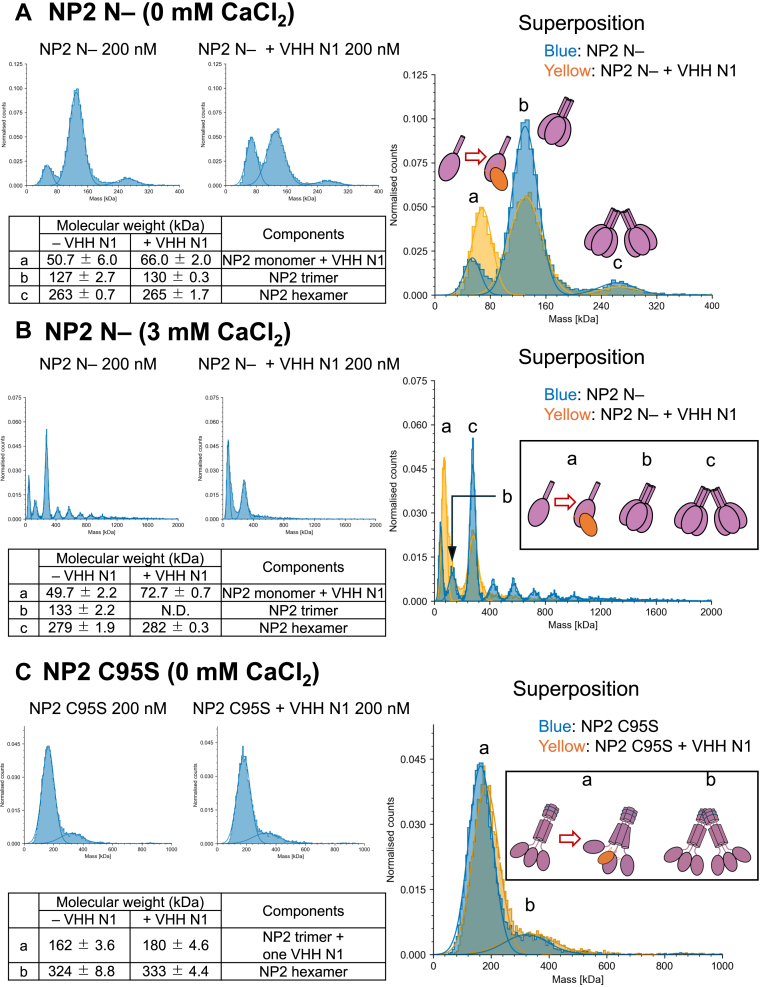
**VHH N1 inhibited the oligomerization of NP2 and increased the ratio of NP2 monomer**. *A*, evaluation using NP2 N- in the absence of CaCl_2_. *B*, evaluation using NP2 N- in the presence of CaCl_2_. *C*, evaluation using NP2 C95S in the absence of CaCl_2_. Equal amounts of VHH N1 and NP2 were mixed at a final concentration of 200 nM (calculated as monomer) and then subjected to analyses using mass photometry. Measurements were conducted using a buffer composed of 10 mM Hepes–NaOH (pH 7.4) and 150 mM NaCl with or without 3 mM CaCl_2_ at 25 °C. Each measurement was performed three times independently. NP, neuronal penntraxin; VHH, variable domain of a heavy-chain antibody.

We found that NP2 highly oligomerizes *via* the N-terminal CC region in the presence of Ca^2+^ (Fig. 1, *C* and *E*). Therefore, we used MP to evaluate the effect of VHH N1 on the clustering of NP2. Equimolar VHH N1 and NP2 N- were mixed (the concentration of NP2 N- was calculated as a monomer) and incubated for several minutes. In the absence of Ca^2+^, a peak corresponding to trimerized NP2 N- decreased and a peak corresponding to the one-to-one complex of NP2 N- and VHH N1 appeared (Fig. 7*A*). Intriguingly, a peak corresponding to the complex of trimerized NP2 N- and VHH N1 was not observed (Fig. 7*A*), suggesting that VHH N1 destroyed the NP2 N- trimer.

Subsequently, we examined the inhibitory effect of VHH N1 in the presence of Ca^2+^. The peaks derived from oligomers larger than hexamers were significantly reduced (Fig. 7*B*). Given that NP2 oligomerizes *via* the N-terminal side (Fig. 1, *C*–*E*), VHH N1 may indirectly inhibit NP2 oligomerization by binding to the PTX domain and causing steric hindrance between the adjacent protomers.

**Figure 8:**
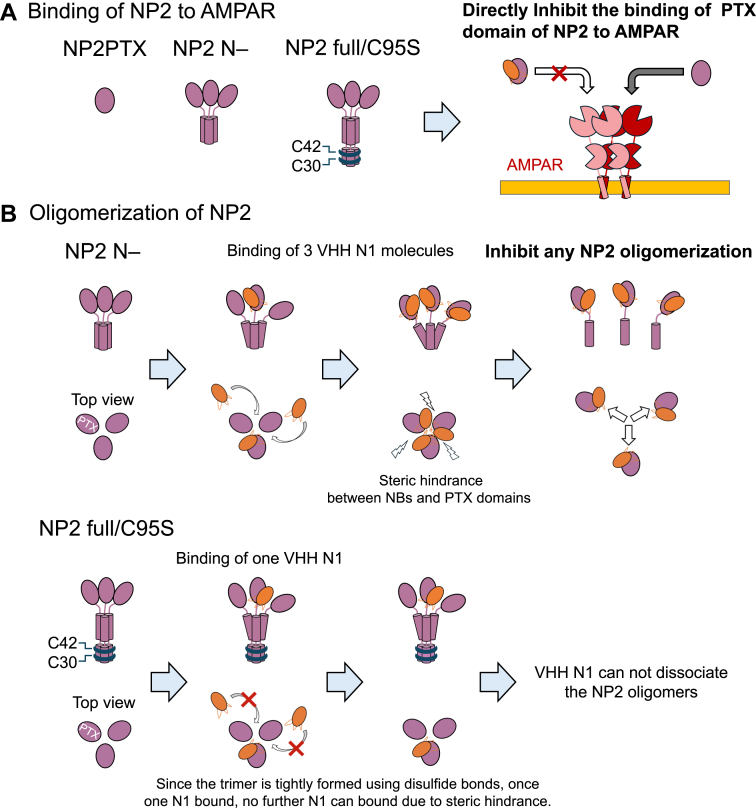
**Inhibitory mechanism of VHH N1 against the functions of NP2**. *A*, VHH N1 directly inhibited the binding of the PTX domain of NP2 to AMPARs. *B*, VHH N1 potentially has the ability to interfere with NP2 multimer formation by binding to the gap between adjacent PTX domains in the NP2 multimer, causing steric hindrance between PTX domains but cannot inhibit the formation of full-length NP2 aggregates that are tightly formed by multiple disulfide bonds. AMPAR, amino-3-hydroxy-5-methyl-4-isoxazolepropionic acid–type receptor; NP, neuronal pentraxin; PTX, pentraxin; VHH, variable domain of a heavy-chain antibody.

To examine the disassembley activity of VHH N1 on full-length NP2, which forms disulfide bonds *via* the CC1 region, we evaluated the effect of VHH N1 against NP2 C95S using the same procedure. The results showed that VHH N1 did not disassemble the NP2 trimer, probably because NP2 C95S forms a strong trimer *via* disulfide bonds. Meanwhile, whereas VHH N1 bound three molecules to the NP2 N- trimer, only one molecule of VHH N1 bound to NP2 C95S. Taken together with the results that VHH N1 disassembled the oligomer of NP2 N-, it is suggested that VHH N1 cannot bind to three protomers of NP2 simultaneously because of the steric hindrance (Fig. 8). In the case of NP2 N-, the binding affinity of VHH N1 prevailed over the interaction between NP2 protomers, thus VHH N1 disassembles the NP2 N- trimer. In contrast, as for NP2 C95S, the trimer linked *via* the S-S bond in CC1 would be strong enough to prevent the binding of VHH N1 molecules and subsequent monomerization. This suggests that the VHH N1 binding interface in PTX would align with the trimer interface in full-length NP2 (Fig. 8).

### Inhibitory activity of VHH N1 at the cell surface

**Figure 9:**
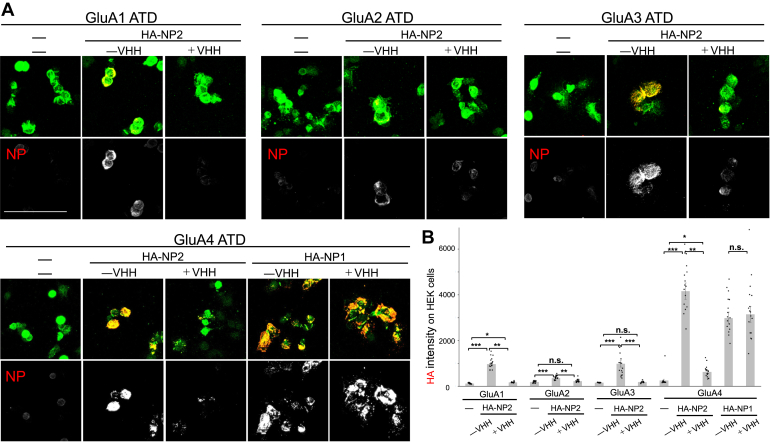
**Inhibition of the interaction between HA-NP2 or HA-NP1 and ATDs of GluAs by VHH in the cell-based binding assay**. *A*, representative cell images. *B*, the graphs show mean HA immunoreactivity in the myc-positive area. The graph represents means ± SEM from two independent experiments, each based on eight or nine fields of view. All data points are shown as *dots*. Statistical significance between groups was assessed using the Kruskal–Wallis test followed by Scheffé's post hoc test or Student’s *t* test. ∗∗∗*p* < 0.001, ∗∗*p* < 0.01, ∗*p* < 0.05, ns, no significance, *p* > 0.05. ATD, amino-terminal domain; HA, hemagglutinin; NP, neuronal pentraxin; VHH, variable domain of a heavy-chain antibody.

Finally, we evaluated the inhibitory effect of VHH N1 on the binding of full-length NP2 to the GluA receptor on the cell surface. The ATD region of GluA1–4 was expressed on the cell surface, and cell supernatants expressing full-length NP1 or NP2 proteins were added in the presence or the absence of VHH N1. In the absence of VHH N1, NP2 strongly bound to GluA4 and also interacted with GluA1 and GluA3 (Fig. 9). The binding signals of NP2 were significantly reduced in the presence of VHH N1, indicating that VHH N1 inhibits the binding of NP2 PTX to GluA receptors (Fig. 9). In contrast, NP1 maintained its binding activity to GluA4, regardless of the addition of VHH N1, consistent with the high specificity of VHH N1 for NP2. Collectively, these results demonstrate that VHH N1 specifically inhibits the binding of NP2 to GluA on the cell surface.

## Discussion

In this study, we generated a nanobody (VHH N1) that specifically recognizes the PTX domain of NP2 and inhibits both the binding of full-length NP2 to GluA and the oligomerization of NP2 N-. This is the first report of a functional nanobody targeting NP2. Although the precise binding mechanism of NP2 to GluA has not been elucidated at the atomic level, the inhibitory effect of VHH N1 suggests that the GluA binding site is located near the VHH N1 binding site. Furthermore, given the high sequence homology between NP1 and NP2, NP1 likely binds to GluA through a similar molecular recognition mechanism. The ability of VHH N1 to inhibit oligomerization of CC1-lacking NP2 provides us with the molecular mechanism of NP2 oligomerization; more specifically, the information about the oligomerization interface. Our results suggest that in full-length NP2, the VHH N1 binding interface in PTX would align with the trimer interface, which may provide important insight into the mechanism of the oligomerization of NP2.

We showed that NP2 forms a trimer *via* CC2 and oligomerizes *via* the N-terminal region in the absence of Ca^2+^. Previous studies suggested that NPs are symmetrically aligned on a hexagon (21) or that the trimer forms a dimer (24), and the hexametric structure of NPs has not been confirmed. Our results suggest that oligomerization proceeds with the trimer as the smallest unit and supports the latter model. Furthermore, although NP2 N- formed larger assemblies in the presence of Ca^2+^, the molecular size increased by trimer units, indicating that NP2 methodically assembled rather than aggregated. The crystal structure of NP1 PTX showed that the PTX domain has two Ca^2+^ binding sites (24), and the NP2 PTX structure determined in this study in complex with VHH N1 revealed that NP2 possesses Ca^2+^ binding sites at positions corresponding to the NP1 sites. Taken together with our results showing that NP2 N- formed larger complexes in the presence of Ca^2+^, the data indicate that binding of Ca^2+^ to this site may play a role in NP assembly. Moreover, considering that CC2 alone shows Ca^2+^-independent association, whereas NP2 N- shows Ca^2+^-dependent association, it is likely that PTX domain itself does not directly oligomerize but indirectly regulates the Ca^2+^-dependent oligomerization of NP2, although we cannot exclude the possibility that PTX domain also has an ability to oligomerize in the presence of Ca^2+^ and it emerge only when linked to a strong self-associating domain such as CC region. Although the biological significance of Ca^2+^-dependent NP2 clustering needs to be elucidated further, our results, together with those showing that NPs bind to GluA in the presence of Ca^2+^ (24) indicate that the clustering may be important in the quantitative control of GluA-mediated synapse formation.

X-ray crystallography analysis revealed that CDR3 of VHH N1 characteristically contributes little to the interaction interface. In addition, compared with conventional VHH, the CDR3 of VHH N1 is very short (35). VHH N1 also has a very unique molecular recognition mechanism in that it surrounds the protruding W419 of NP2. In contrast, the molecular recognition mechanism often observed in VHHs allows CDR3 to enter the concave surface of the antigen (36). Although molecular recognition by a VHH that tightly surrounds the tryptophan residue has been reported previously, the recognition mechanism of VHH N1 differs greatly from it. The VHH reported in the previous study used a long CDR3 loop composed of more than 20 residues and a hydrophobic interaction as the core (37), whereas VHH N1 used hydrophobic and hydrophilic residues from both FR2 and CDR3 to recognize the tryptophan residue. Thus, VHH N1 has a novel molecular recognition mechanism, which contributes to our understanding of the diversity and utility of molecular recognition mechanisms by VHHs.

VHH N1 bound to NP2 and showed inhibitory activities at the cell surface. Taken together with the results showing that VHH N1 did not disassemble the oligomer formed by the full length of NP2, the VHH N1 would inhibit the binding of NP2 to AMPAR without changing the oligomerization state. Given that VHH N1 recognizes NP2 with high specificity, this VHH can be applied to detect NP2, regardless of the presence of NP1 and NPR and to regulate NP2 activity *in vivo*. For example, VHH N1 may be used as a diagnostic agent for AD, as NP2 is downregulated in AD patients and is attracting attention as a marker molecule (26, 27). In addition, previous studies suggested that overexpression of NP2 in peripheral nerves contributes to itchiness, mainly chronic itch (28, 29). Therefore, VHH N1 can be applied to the development of therapeutic agents to alleviate symptoms by suppressing NP2 function, which is a fundamentally different mechanism from those of conventional topical steroids and antihistamine drugs.

In summary, we demonstrated that Ca^2+^-dependent clustering of NP2 occurs *via* the CC region. We generated a VHH that specifically recognizes NP2 and inhibits receptor binding. In addition, X-ray crystallographic analysis elucidated the characteristic molecular recognition mode of the VHH. Our findings provide important insights into the molecular mechanism underlying NP2 function and highlight the potential of VHH N1 for therapeutic applications.

## Experimental procedures

### Expression and purification of Nptxs

All NP constructs (NP2 full, NP2 C95S, NP2 N-, NP1 PTX, NP2 PTX, NPR PTX, and NP2 CC2) were expressed using the same method. The DNA sequence encoding human NPs with His-tag at the C terminus was cloned into the pcDNA 3.4 vector (A14697; Thermo Fisher Scientific). Recombinant NP proteins were expressed using Expi293 cells (A14635; Thermo Fisher Scientific) following the manufacturer’s methods. The cells were cultured for 6 days after transfection at 37 °C and 8% CO_2_.

Purification methods were identical for all constructs. The supernatant was collected and filtered through a 0.8 μm pore-sized filter, followed by dialysis against a buffer composed of 20 mM Tris–HCl (pH 8.0), 500 mM NaCl, and 5 mM imidazole. The sample was purified by immobilized metal affinity chromatography (IMAC) using Ni–NTA Agarose (30230; Qiagen), and the proteins were eluted with 20 mM Tris–HCl (pH 8.0), 500 mM NaCl, and 200 mM imidazole. The proteins were dialyzed against a solution composed of 10 mM Hepes–NaOH (pH 7.4) and 150 mM NaCl. Final purification was performed by SEC using a HiLoad 16/60/20 Superdex 200 pg column (28989335; Cytiva) at 4 °C equilibrated in buffer containing 10 mM Hepes–NaOH (pH 7.4) and 150 mM NaCl. The purity was confirmed by SDS-PAGE.

### MP analysis

The oligomerization states of NPs were evaluated using the Two^MP^ system (Refeyn) at 25 °C. Samples were dialyzed against buffer containing 10 mM Hepes–NaOH (pH 7.4) and 150 mM NaCl with and without 3 mM CaCl_2_. The final concentration of NPs was 200 nM. The molecular weights of the samples were calculated using DiscoverMP software (Refeyn).

### Immunization and library construction

We immunized an alpaca with the recombinant NP2 N- protein. Following immunization, a blood sample was collected, and lymphocytes were separated. Total RNA was extracted using Trizol (Thermo Fisher Scientific) according to the manufacturer’s protocol. Following RNA extraction, complementary DNA was synthesized by reverse transcription using the Superscript 3 First-Strand kit (Thermo Fisher Scientific).

To generate the alpaca VHH Library, the variable region of the heavy-chain antibodies was amplified from complementary DNA by PCR using KOD One PCR Master Mix (TOYOBO). Amplified fragments were inserted into the phagemid vector pLUCK (38), and the phagemid vectors were purified and desalted using AMPure XP (Beckman Coulter). Next, 320 ng of purified DNA were introduced into TG1-competent cells (Lucigen) by electroporation. The size of the resulting library was 1.7 × 10^8^.

### Selection of VHH using phage display

*Escherichia coli* cells harboring the immune library were infected with helper phages and incubated overnight. The phages were precipitated and purified from the supernatant by polyethylene glycol precipitation (39). Purified phages displaying VHHs were added to an immunotube (Thermo Fisher Scientific) coated with 10 μg/ml of NP2 N-, followed by blocking with PBS–0.05% Tween-20 (PBS-T) containing 3% skim milk. The mixture was incubated for 1 h at room temperature. The immunotube was washed 10 times with PBS-T. Bound phages were eluted with 0.1 M glycine–HCl (pH 2.2) and neutralized with 1 M Tris (pH 9.1). Eluted phages were used to infect XL-1 blue *E*. *coli*, followed by infection with helper phages. After overnight incubation, phages were precipitated and used for the next round of panning. After the third round, phage-infected *E*. *coli* cells were plated. Forty-six individual colonies were randomly picked, and phages were produced from each colony for screening using ELISA against NP2 N-.

For screening by ELISA, a 96-well immunosorbent plate was coated with 10 μg/ml of NP2 N- and blocked with PBS-T containing 5% skim milk. The 46 phage clones were applied to the plate and incubated for 1 h at room temperature. After washing three times with PBS-T, 2000 times diluted rabbit serum immunized with recombinant gene 8 of the M13 phage was applied, and the mixture was incubated for 1 h at room temperature. After washing three times with PBS-T, 5000 times diluted anti-rabbit immunoglobulin G (IgG) horseradish peroxidase–linked antibody was added, and the mixture was incubated for 1 h at room temperature. After wells were washed three times with PBS-T, 3,3′,5,5′-tetramethylbenzidine (ScyTek) was added for colorimetric quantification. After 30 min of incubation at room temperature, reactions were stopped using 3,3′,5,5′-tetramethylbenzidine stop buffer (ScyTek), and absorbance was read at 450 nm using a PHERAstar Plus microplate reader (BMG).

### Expression and purification of VHH N1

The DNA sequence encoding VHH N1 with His-tag at the C terminus was cloned into the pRA2 vector (40). VHHs were expressed using BL21 (DE3) *E*. *coli* (Merck). Next, 0.5 mM isopropyl-1-thio-β-D-galactopyronoside was added to the growing cells when the absorbance at 600 nm reached 0.9 to 1.1 to induce expression of proteins, and an overnight culture was conducted. The *E*. *coli* pellet was resuspended in 20 mM Tris–HCl (pH 8.0), 500 mM NaCl, and 5 mM imidazole, and then the cells were disrupted by sonication. The expressed protein was purified by IMAC using Ni–NTA agarose (30230; Qiagen). The proteins were eluted with 20 mM Tris–HCl (pH 8.0), 500 mM NaCl, and 200 mM imidazole. The proteins were dialyzed against buffer containing 10 mM Hepes–NaOH (pH 7.4) and 150 mM NaCl. Final purification was performed by SEC using a HiLoad 16/60/20 Superdex 75 pg column (28989333; Cytiva) at 4°C equilibrated in buffer containing 10 mM Hepes–NaOH (pH 7.4) and 150 mM NaCl. The purity was confirmed by SDS-PAGE.

### SPR analyses of the interaction between VHH N1 and NPs

The kinetic parameters of interactions between NP constructs and VHH N1 were determined using a Biacore 8K instrument (Cytiva). NP constructs were immobilized on a CM5 Biacore sensor chip (29149604; Cytiva) at around 800 resonance units (RUs) for NP2 N- and 600 RU for NP1 PTX, NP2 PTX, and NPR PTX using amine-coupling methods according to the manufacturer’s manual. VHH N1 was injected into the sensor chip at a flow rate of 30 μl/min. The range of concentration of VHH N1 was 1.25, 2.5, 5, 10, and 20 nM against NP2 N- and NP2 PTX for the analyses in the presence of Ca^2+^; 5, 10, 20, 40, and 80 nM for the analyses in the absence of Ca^2+^; and 625, 1250, 2500, 5000, and 10,000 nM against NP1 PTX and NPR PTX. The association time was 120 s for all measurements; dissociation time was 600 s for the cases of NP2 N- and NP2 PTX and 240 s for the cases of NP1 PTX and NPR PTX. The assays were carried out with the buffer containing 10 mM Hepes–NaOH (pH 7.4), 150 mM NaCl, and Tween-20 (0.005%) with and without 3 mM CaCl_2_. The data were collected using the single-cycle kinetics method for NP2 N- and NP2 PTX and using the multicycle kinetics method for NP1 PTX and NPR PTX. The data were analyzed using BIA evaluation software (Cytiva).

### ITC analyses of the interaction between VHH N1 and NPs

The thermodynamic parameters of interactions between NP2 PTX and VHH N1 were evaluated using an iTC200 microcalorimeter (Malvern Panalytical). Samples were dialyzed against buffer containing 10 mM Hepes–NaOH (pH 7.4) and 150 mM NaCl with or without 3 mM CaCl_2_. Fifty micromolars of NP2 PTX were titrated from the syringe into a cell filled with 5 μM VHH N1. The thermodynamic parameters were calculated by fitting of titration curve using ORIGIN 7.0 software (Malvern Panalytical).

### VHH N1 binding assay using intact cells

HEK293 cells were transfected with HA-tagged mouse full-length NP1 (HA-NP1), full-length HA-tagged NP2 (HA-NP2), or the extracellular domain of NPR (HA-NPR), and 48 h after transfection, the cells were fixed with 4% paraformaldehyde, permeabilized, and immunostained with mouse anti-HA (mouse; Covance) and VHH N1 (1 μg/ml). They were immunostained with rabbit anti-His (Cell Signaling). Alexa Fluor 488–conjugated donkey anti-mouse IgG (H + L) (1:1000 dilution; Molecular Probe) and Cy3-conjugated donkey anti-rabbit IgG (H + L) (1:1000 dilution; Jackson Laboratory) were used as secondary antibodies. Images were taken with a confocal laser-scanning microscope (FV1000; Olympus).

### Crystallization

Purified NP2 PTX and VHH N1 were mixed at a ratio of 1:1.2, concentrated to about 1 mg/ml (40 μM) using Amicon ultra-4 10K, followed by concentration using Amicon Ultra-4 30K (Merck Millipore) to simultaneously remove excess VHH N1, and finally concentrated to 10 mg/ml (400 μM) in buffer containing 10 mM Hepes–NaOH (pH 7.4) and 150 mM NaCl. Crystallization screening was performed using an Oryx8 protein crystallization robot (Douglas Instruments) with commercial screening kits (PEG/Ion 1/2 [Hampton Research]) by vapor diffusion following the sitting drop method. The crystals of the complex of NP2 PTX and VHH N1 were obtained in solution containing 1% w/v tryptone, 0.001 M sodium azide, 0.05 M Hepes–sodium (pH 7.0), and 12% w/v PEG 3350. Suitable crystals were harvested, incubated in a solution containing mother liquor supplemented with 25% glycerol, and subsequently transferred to liquid nitrogen for storage until data collection.

### Data collection and refinement

Diffraction data from a single crystal of the NP2 PTX and VHH N1 complex were collected in beamline AR-NW12A at the Photon Factory (Tsukuba) under cryogenic conditions (100 K). Diffraction images were processed using the program XDS (41) and merged and scaled with the program AIMLESS (42) of the CCP4 suite (43). The structure of the WT protein was determined by the molecular replacement method using the predicted model of unbound NP2 PTX and unbound VHH N1 using AlphaFold2 (44) and with PHASER (45). The models were refined with the program REFMACS (46) and built manually with COOT (https://www2.mrc-lmb.cam.ac.uk/personal/pemsley/coot/docs/coot-faq.html#Citing) (47). Validation was carried out with MOLPROBITY (48). Data collection and structure refinement statistics are given in Table 3.

**Table 3:** Data collection and refinement statistics

NP2 PTX + N1 VHH	
**Data collection**	
Space group	P 2_1_ 2_1_ 2
Unit cell	
a, b, c (Å)	93.6, 118.2, 39.5
α, β, γ (°)	90.0, 90.0, 90.0
Resolution (Å)	46.8–2.02 (2.07–2.02)
Wavelength	1.0000
Observations	363,197 (16,156)
Unique reflections	29,010 (1714)
*R*_merge_^a^	0.066 (1.051)
*R*_pim_	0.019 (0.353)
CC_1/2_	1.000 (0.782)
*I/σ (I)*	31.7 (2.4)
Multiplicity	12.5 (9.4)
Completeness (%)	98.2 (80.8)
**Refinement statistics**	
Resolution (Å)	46.8–2.02
*R*_work_/*R*_free_ (%)^b^^,^^c^	18.2/22.0
No. of complexes	1
No. of atoms	
N1 VHH	931
NP2 PTX	1633
Other	13
Water	126
*B*-factor (Å^2^)	
N1 VHH	49.8
NP2 PTX	44.7
Other	60.9
Water	43.6
Ramachandran plot^d^	
Preferred (%)	96.0
Allowed (%)	3.7
Outliers (%)	0.3
RMSD bond (Å)	0.007
RMSD angle (°)	1.62
PDB entry code	9LAD

Statistical values given in parentheses refer to the highest resolution bin. The software employed is listed in the Experimental procedures section.

a *R*_merge_ = ∑_hkl_ ∑_i_|I(hkl)_i_-{I(hkl)}|/ ∑∑_hkl_ ∑_i_I(hkl)_i._

b *R*_work_ = ∑_hkl_|F(hkl)_c_- {F(hkl)_o_}|/ ∑_hkl_F(hkl)_o._

c *R*_free_ was calculated exactly as *R*_work_, where F(hkl)_o_ were taken from 5% of the data not included in refinement.

d Values with and without square brackets correspond to values calculated with MOLPROBITY (48). Residue Ser40 of NP2 PTX was found to be an outlier with MOLPROBITY (48).

### Mutational analyses

Single alanine mutants of NP2 PTX were expressed and purified by the same procedure as those used in NP2 PTX WT. The kinetic parameters of interactions between NP2 mutants and VHH N1 were determined using a Biacore 8K instrument (Cytiva). Each NP2 mutant was immobilized on a CM5 Biacore sensor chip (29149604; Cytiva) at around 800 Rus using amine-coupling methods according to the manufacturer’s manual. VHH N1 was injected into the sensor chip at a flow rate of 30 μl/min. The range of concentration of VHH N1 was 1.25, 2.5, 5, 10, and 20 nM against D407A_NP2 PTX_, 62.5, 125, 250, 500, and 1000 nM for V412A_NP2 PTX_ and W419A_NP2 PTX_. The association time was 120 s, and the dissociation time was 600 s for all measurements. The assays were carried out with buffer containing 10 mM Hepes–NaOH (pH 7.4), 150 mM NaCl, and Tween-20 (0.005%) with 3 mM CaCl_2_.

### Expression and purification of GluA4

The DNA sequence encoding the human GluA4 ATD with His-tag at the C terminus was cloned into pcDNA 3.4 vector (A14697; Thermo Fisher Scientific). Recombinant GluA4 was expressed using Expi293 cells (A14635; Thermo Fisher Scientific) following the manufacturer’s methods. The cells were cultured for 5 days after transfection at 37°C and 8% CO_2_.

The supernatant was collected and filtered, followed by dialysis against a solution composed of 20 mM Tris–HCl (pH 8.0), 500 mM NaCl, and 5 mM imidazole. The samples were purified by IMAC using Ni–NTA Agarose (30230; Qiagen), and the proteins were eluted with 20 mM Tris–HCl (pH 8.0), 500 mM NaCl, and 200 mM imidazole. The proteins were dialyzed against a solution composed of 10 mM Hepes–NaOH (pH 7.4), 150 mM NaCl, and 3 mM CaCl_2_. Final purification was performed by SEC using a HiLoad 16/600 Superdex 200 pg column (28989335; Cytiva) at 4 °C equilibrated in buffer containing 10 mM Hepes–NaOH (pH 7.4), 150 mM NaCl, and 3 mM CaCl_2_.

### Interaction analyses between NP2 and GluA4

The assays were performed using a Biacore T200 instrument (Cytiva). The ATD domains of GluA4 were immobilized on a CM5 Biacore sensor chip (29149604; Cytiva) at around 400 RU, using amine-coupling methods according to the manufacturer’s methods. NP2 constructs were injected into the sensor chip at a flow rate of 30 μl/min. The range of concentration of NP2 was from 31.25 to 2000 nM in the case of NP2 N- and NP2 PTX and from 62.5 to 4000 nM in the case of NP2 CC2. The association time was 200 s, and the dissociation time was 300 s. The assays were carried out with the buffer containing 10 mM Hepes––NaOH (pH 7.4), 150 mM NaCl, and Tween-20 (0.005%) with and without 3 mM CaCl_2_.

### Inhibitory analyses of VHH N1 on the binding of NP2 to the GluA receptor

The assays were performed using a Biacore T200 instrument. The ATD domains of GluA4 were immobilized on a CM5 Biacore sensor chip (29149604; Cytiva) at around 800 RU, using amine-coupling methods according to the manufacturer’s methods. The complex of NP2 PTX and VHH N1 was injected to evaluate the binding activity. NP2 PTX (500 nM) was mixed with 0 to 1000 nM of VHH N1. The association time was 200 s, and the dissociation time was 300 s for all measurements. The assays were carried out with buffer containing 10 mM Hepes–NaOH (pH 7.4), 150 mM NaCl, 3 mM CaCl_2_, and 0.005% Tween-20.

### Analyses of VHH N1 inhibition of oligomerization of NP2

The oligomerization states of the complex of NP2 N- and VHH N1 were evaluated using Two^MP^ (Refeyn) at 25 °C. Samples were dialyzed against buffer containing 10 mM Hepes–NaOH (pH 7.4) and 150 mM NaCl with or without 3 mM CaCl_2_. The final concentrations of NP2 N-, NP2 C95S, and VHH N1 were 200 nM, respectively. The molecular weights of the samples were calculated using DiscoverMP software.

### NP binding assay using intact cells

HEK293 cells 48 h after transfection with pDisplay myc-tagged GluA-ATD (24) were incubated with HA-NP2 (0.25 nM) or HA-NP1 (0.68 nM) with or without VHH for NP2 (3.35 μM) for 4 h, fixed with 4% paraformaldehyde, and immunostained with mouse anti-HA (mouse; Covance) in a nonpermeable condition to selectively stain HA-NP2 or HA-NP1 on the cell surface. After permeabilization, cells were stained with rabbit anti-myc antibody (MBL Life Sciences), followed by Cy3-conjugated donkey anti-mouse IgG (H + L) (1:1000 dilution; Jackson Laboratory) and Alexa Fluor 488–conjugated donkey anti-rabbit IgG (H + L) secondary antibodies (1:1000 dilution; Molecular Probe). Images were captured using a CCD camera (Zyla 4.2; Andor) attached to a fluorescence microscope (BX63; Olympus) in at least eight randomly selected fields of view (each field corresponded to 665.6 × 665.6 μm) using a fixed gain and exposure time for each fluorescent channel. The images were semiautomatically analyzed using ImageJ software (http://imagej.nih.gov/ij/). Regions of interest (ROIs) of HEK293 cells expressing receptors were determined by the signal intensity and area size fulfilling the threshold (Huang) and particle definition. The signal intensity within the ROI was determined by subtracting the mean signal intensity outside the ROI for each field.

### Constructs for *Mus musculus* NP proteins

Constructs for *Mus musculus* NP2 proteins (amino acid residues: 15–429; GenBank: BC026054.1), NP1 proteins (amino acid residues: 23–432; GenBank: NM_008730.2 with one silent mutation c321t), and NPR (amino acid residues: 24–493; GenBank: NM_030689.4. nucleotides 90–513 are codon-optimized.) were amplified by PCR and cloned into pCAGGS vector (kindly provided by Dr J. Miyazaki, Osaka University, Osaka, Japan) to introduce an N-terminal Igκ secretion signal sequence together with HA-tag.

HA-tagged NP1 or NP2 proteins were expressed in HEK293 tSA cells (a kind gift from Dr R. Horn, Thomas Jefferson University Medical School, Philadelphia, PA) as described previously (49). The concentration of each recombinant HA-tagged NP1 or NP2 was quantified by immunoblot analysis using anti-HA antibody (mouse; Covance) with purified histidine plus HA-tagged Cbln4 (2 mg/ml) as the standard.

## Data availability

The coordinates and structure factors of the complex between the nanobody NP2 PTX and VHH N1 have been deposited in the PDB with entry code 9LAD.

## Supporting information

This article contains supporting information.

## Conflict of interests

The authors declare that they have no conflicts of interest with the contents of this article.

## References

[1] Azevedo F.A.C., Carvalho L.R.B., Grinberg L.T., Farfel J.M., Ferretti R.E.L., Leite R.E.P. (2009). Equal numbers of neuronal and nonneuronal cells make the human brain an isometrically scaled-up primate brain. J. Comp. Neurol..

[2] Nelson S.B., Valakh V. (2015). Excitatory/inhibitory balance and circuit homeostasis in autism spectrum disorders. Neuron.

[3] Palop J.J., Mucke L. (2016). Network abnormalities and interneuron dysfunction in Alzheimer disease. Nat. Rev. Neurosci..

[4] Bozzi Y., Provenzano G., Casarosa S. (2018). Neurobiological bases of autism-epilepsy comorbidity: a focus on excitation/inhibition imbalance. Eur. J. Neurosci..

[5] Foss-Feig J.H., Adkinson B.D., Ji J.L., Yang G., Srihari V.H., McPartland J.C. (2017). Searching for cross-diagnostic convergence: neural mechanisms governing excitation and inhibition balance in schizophrenia and autism spectrum disorders. Biol. Psychiatry.

[6] Forrest M.P., Parnell E., Penzes P. (2018). Dendritic structural plasticity and neuropsychiatric disease. Nat. Rev. Neurosci..

[7] Yuzaki M. (2018). Two classes of secreted synaptic organizers in the central nervous system. Annu. Rev. Physiol..

[8] Südhof T.C. (2018). Towards an understanding of synapse formation. Neuron.

[9] Jang S., Lee H., Kim E. (2017). Synaptic adhesion molecules and excitatory synaptic transmission. Curr. Opin. Neurobiol..

[10] Südhof T.C. (2017). Synaptic neurexin complexes: a molecular code for the logic of neural circuits. Cell.

[11] Dabrowski A., Terauchi A., Strong C., Umemori H. (2015). Distinct sets of FGF receptors sculpt excitatory and inhibitory synaptogenesis. Development.

[12] Taetzsch T., Brayman V.L., Valdez G. (2018). FGF binding proteins (FGFBPs): modulators of FGF signaling in the developing, adult, and stressed nervous system. Biochim. Biophys. Acta Mol. Basis Dis..

[13] Teo S., Salinas P.C. (2021). Wnt-Frizzled signaling regulates activity-mediated synapse formation. Front. Mol. Neurosci..

[14] Johnson-Venkatesh E.M., Umemori H. (2010). Secreted factors as synaptic organizers. Eur. J. Neurosci..

[15] Dingledine R., Borges K., Bowie D., Traynelis S.F. (1999). The glutamate receptor ion channels. Pharmacol. Rev..

[16] Martinez de la Torre Y., Fabbri M., Jaillon S., Bastone A., Nebuloni M., Vecchi A. (2010). Evolution of the pentraxin family: the new entry PTX4. J. Immunol..

[17] Schlimgen A., Helms J.A., Vogel H., Perin M.S. (1995). Neuronal pentraxin, a secreted protein with homology to acute phase proteins of the immune system. Neuron.

[18] Dodds D.C., Omeis I.A., Cushman S.J., Helms J.A., Perin M.S. (1997). Neuronal pentraxin receptor, a novel putative integral membrane pentraxin that interacts with neuronal pentraxin 1 and 2 and taipoxin-associated calcium-binding protein 49. J. Biol. Chem..

[19] Tsui C., Copeland N., Gilbert D., Jenkins N., Barnes C., Worley P. (1996). Narp, a novel member of the pentraxin family, promotes neurite outgrowth and is dynamically regulated by neuronal activity. J. Neurosci..

[20] Cho R.W., Park J.M., Wolff S.B.E., Xu D., Hopf C., Kim J.-A. (2008). mGluR1/5-dependent long-term depression requires the regulated ectodomain cleavage of neuronal pentraxin NPR by TACE. Neuron.

[21] Xu D., Hopf C., Reddy R., Cho R.W., Guo L., Lanahan A. (2003). Narp and NP1 form heterocomplexes that function in developmental and activity-dependent synaptic plasticity. Neuron.

[22] O’Brien R.J., Xu D., Petralia R.S., Steward O., Huganir R.L., Worley P. (1999). Synaptic clustering of AMPA receptors by the extracellular immediate-early gene product narp. Neuron.

[23] O’Brien R., Xu D., Mi R., Tang X., Hopf C., Worley P. (2002). Synaptically targeted narp plays an essential role in the aggregation of AMPA receptors at excitatory synapses in cultured spinal neurons. J. Neurosci..

[24] Suzuki K., Elegheert J., Song I., Sasakura H., Senkov O., Matsuda K. (2020). A synthetic synaptic organizer protein restores glutamatergic neuronal circuits. Science.

[25] Kirkpatrick L.L., Matzuk M.M., Dodds D.C., Perin M.S. (2000). Biochemical interactions of the neuronal pentraxins. Neuronal pentraxin (NP) receptor binds to taipoxin and taipoxin-associated calcium-binding protein 49 via NP1 and NP2. J. Biol. Chem..

[26] Watson C.M., Dammer E.B., Ping L., Duong D.M., Modeste E., Carter E.K. (2023). Quantitative mass spectrometry analysis of cerebrospinal fluid protein biomarkers in Alzheimer’s disease. Sci. Data.

[27] Nilsson J., Pichet Binette A., Palmqvist S., Brum W.S., Janelidze S., Ashton N.J. (2024). Cerebrospinal fluid biomarker panel for synaptic dysfunction in a broad spectrum of neurodegenerative diseases. Brain.

[28] Kanehisa K., Koga K., Maejima S., Shiraishi Y., Asai K., Shiratori-Hayashi M. (2022). Neuronal pentraxin 2 is required for facilitating excitatory synaptic inputs onto spinal neurons involved in pruriceptive transmission in a model of chronic itch. Nat. Commun..

[29] Wang R., Man Y., Zhou M., Zhu Y., Wang L., Yang J. (2021). Neuropathic pain-induced cognitive dysfunction and down-regulation of neuronal pentraxin 2 in the cortex and hippocampus. Neuroreport.

[30] Arbabi-Ghahroudi M. (2017). Camelid single-domain antibodies: historical perspective and future outlook. Front. Immunol..

[31] Zuber B., Nikonenko I., Klauser P., Muller D., Dubochet J. (2005). The mammalian central nervous synaptic cleft contains a high density of periodically organized complexes. Proc. Natl. Acad. Sci. U. S. A..

[32] Kuroda D., Tsumoto K. (2023). Structural classification of CDR-H3 in single-domain VHH antibodies. Methods Mol. Biol..

[33] Xiao M.-F., Xu D., Craig M.T., Pelkey K.A., Chien C.-C., Shi Y. (2017). NPTX2 and cognitive dysfunction in Alzheimer’s disease. Elife.

[34] Pelkey K.A., Barksdale E., Craig M.T., Yuan X., Sukumaran M., Vargish G.A. (2015). Pentraxins coordinate excitatory synapse maturation and circuit integration of parvalbumin interneurons. Neuron.

[35] Nakakido M., Kinoshita S., Tsumoto K. (2024). Development of novel humanized VHH synthetic libraries based on physicochemical analyses. Sci. Rep..

[36] De Genst E., Silence K., Decanniere K., Conrath K., Loris R., Kinne J. (2006). Molecular basis for the preferential cleft recognition by dromedary heavy-chain antibodies. Proc. Natl. Acad. Sci. U. S. A..

[37] Yokoo T., Tanabe A., Yoshida Y., Caaveiro J.M.M., Nakakido M., Ikeda Y. (2022). Antibody recognition of complement factor H reveals a flexible loop involved in atypical hemolytic uremic syndrome pathogenesis. J. Biol. Chem..

[38] Maenaka K., Furuta M., Tsumoto K., Watanabe K., Ueda Y., Kumagai I. (1996). A stable phage-display system using a phagemid vector: phage display of hen egg-white lysozyme (HEL), Escherichia coli alkaline, phosphatase, and anti-HEL monoclonal antibody, HyHEL10. Biochem. Biophys. Res. Commun..

[39] Lee C.M.Y., Iorno N., Sierro F., Christ D. (2007). Selection of human antibody fragments by phage display. Nat. Protoc..

[40] Makabe K., Asano R., Ito T., Tsumoto K., Kudo T., Kumagai I. (2005). Tumor-directed lymphocyte-activating cytokines: refolding-based preparation of recombinant human interleukin-12 and an antibody variable domain-fused protein by additive-introduced stepwise dialysis. Biochem. Biophys. Res. Commun..

[41] Kabsch W. (2010). Integration, scaling, space-group assignment and post refinement. Acta Crystallogr. D Biol. Crystallogr..

[42] Evans P.R., Murshudov G.N. (2013). How good are my data and what is the resolution?. Acta Crystallogr. D Biol. Crystallogr..

[43] Winn M.D., Ballard C.C., Cowtan K.D., Dodson E.J., Emsley P., Evans P.R. (2011). Overview of the CCP4 suite and current developments. Acta Crystallogr. D Biol. Crystallogr..

[44] Mirdita M., Schütze K., Moriwaki Y., Heo L., Ovchinnikov S., Steinegger M. (2022). ColabFold: making protein folding accessible to all. Nat. Methods.

[45] McCoy A.J., Grosse-Kunstleve R.W., Adams P.D., Winn M.D., Storoni L.C., Read R.J. (2007). Phaser crystallographic software. J. Appl. Crystallogr..

[46] Murshudov G.N., Vagin A.A., Dodson E.J. (1997). Refinement of macromolecular structures by the maximum-likelihood method. Acta Crystallogr. D Biol. Crystallogr..

[47] Emsley P., Lohkamp B., Scott W.G., Cowtan K. (2010). Features and development of coot. Acta Crystallogr. D Biol. Crystallogr..

[48] Chen V.B., Arendall W.B., Headd J.J., Keedy D.A., Immormino R.M., Kapral G.J. (2010). MolProbity: all-atom structure validation for macromolecular crystallography. Acta Crystallogr. D Biol. Crystallogr..

[49] Matsuda K., Budisantoso T., Mitakidis N., Sugaya Y., Miura E., Kakegawa W. (2016). Transsynaptic modulation of kainate receptor functions by C1q-like proteins. Neuron.

