# Supplementary material for: Development of a VHH that inhibits the binding of neuronal pentraxin 2 to a postsynaptic glutamate receptor, AMPAR
Source: J Biol Chem. 2025 Nov 25;302(1):110975. doi: 10.1016/j.jbc.2025.110975 (PMC12799932; doi:10.1016/j.jbc.2025.110975)
Supplement: Supplementary Material 1 [file mmc1.pdf]

>NP2 C95S
AGRAQDSPIPGSRFVCTALPPEAARAGCPLPAMPMQGGALSPEEELRAAVLQLRETVVQQKETLGAQ
REAIRELTGKLAR**S**EGLAGGKARGTGKDTMGDLPRDPGHVVEQLSRSLQTLKDRLESLELQLRTNVS
NAGLPSDFREVLQRRLGELERQLLRKVAELEDEKSLLHNETSAHRQKTESTLNALLQRVTELERGNS
AFKSPDAFKVSLPLRTNYLYGKIKKTLPELYAFTICLWLRSSASPGIGTPFSYAVPGQANEIVLIEW
GNNPIELLINDKVAQLPLFVSDGKWHHICITWTTRDGMWEAFQDGEKLGTGENLAPWHPIKPGGVLI
LGQEQDTVGGRFDATQAFVGELSQFNIWDRVLRAQEIINIANCSTNMPGNIIPWVDNNVDVFGGASK
WPVETCEERLLDL

>NP2 N-
TGKDTMGDLPRDPGHVVEQLSRSLQTLKDRLESLELQLRTNVSNAGLPSDFREVLQRRLGELERQLL
RKVAELEDEKSLLHNETSAHRQKTESTLNALLQRVTELERGNSAFKSPDAFKVSLPLRTNYLYGKIK
KTLPELYAFTICLWLRSSASPGIGTPFSYAVPGQANEIVLIEWGNNPIELLINDKVAQLPLFVSDGK
WHHICITWTTRDGMWEAFQDGEKLGTGENLAPWHPIKPGGVLILGQEQDTVGGRFDATQAFVGELSQ
FNIWDRVLRAQEIINIANCSTNMPGNIIPWVDNNVDVFGGASKWPVETCEERLLDLAAAHHHHHH

>NP2 PTX
GDAFKVSLPLRTNYLYGKIKKTLPELYAFTICLWLRSSASPGIGTPFSYAVPGQANEIVLIEWGNNP
IELLINDKVAQLPLFVSDGKWHHICITWTTRDGMWEAFQDGEKLGTGENLAPWHPIKPGGVLILGQE
QDTVGGRFDATQAFVGELSQFNIWDRVLRAQEIINIANCSTNMPGNIIPWVDNNVDVFGGASKWPVE
TCEERLLDLAAAHHHHHH

>NP2 CC2
TGKDTMGDLPRDPGHVVEQLSRSLQTLKDRLESLELQLRTNVSNAGLPSD
FREVLQRRLGELERQLLRKVAELEDEKSLLHNETSAHRQKTESTLNALLQ
RVTELERGNSAFKSAAAHHHHHH

>NP1 PTX
DKFQLTFPLRTNYMYAKVKKSLPEMYAFTVCMWLKSSAAPGVGTPFSYAVPGQANELVLIEWGNNPM
EILINDKVAKLPFVINDGKWHHICVTWTTRDGVWEAYQDGTQGGNGENLAPYHPIKPQGVLVLGQEQ
DTLGGGFDATQAFVGELAHFNIWDRKLTPGEVYNLATCSSKALSGNVIAWAESQIEIFGGATKWTFE
ACRQINAAAHHHHHH

>NPR PTX
AFKVSIPIRNNYMYARVRKALPELYAFTACMWLRSRSGGSGQGTPFSYSVPGQANEIVLLEAGLEPM
ELLINDKVAQLPLSLKDSNWHHICISWTTRDGLWSAYQDGELRGSGENLAAWHPIKPHGILILGQEQ
DTLGGRFDATQAFVGDIAQFNLWDHALTPAQVLGMANCTGPLMGNVLPWEDKLVEAFGGAKKAAFDV
CKGRAKAAAAHHHHHH

>VHH N1
QLQLVESGGGLVQAGDSLKLSCIDSGSTFNLYAMGWFRQAPGKEREFVASISRGGGSSYYADSQLKG
RFTISRDNAKNTVYLQMNSLKPEDTAVYYCNARAGRYDYWGQGTQVTVSSAAGHHHHHH

>GluA4 ATD
AFPSSVQIGGLFIRNTDQEYTAFRLAIFLHNTSPNASEAPFNLVPHVDNIETANSFAVTNAFCSQYS
RGVFAIFGLYDKRSVHTLTSFCRRLHISLITPSFPTEGESQFVLQLRPSLRGALLSLLDHYEWNCFV
FLYDTDRGYSILQAIMEKAGQNGWHVSAICVENFNDVSYRQLLEELDRRQEKKFVIDCEIERLQNIL
EQIVSVGKHVKGYHYIIANLGFKDISLERFIHGGANVTGFQLVDFNTPMVTKLMDRWKKLDQREYPG
SETPPKYTSALTYDGVLVMAETFRSLRRQKIDISRRGNAGDCLANPAAPWGQGIDMERTLKQVRIQG
LTGNVQFDHYGRRVNYTMDVFELKSTGPRKVGYWNDMDKLVLIQDAAAHHHHHH

Figure. S1 The Amino Acid sequence of NPs, VHH N1, and GluA4ATD. Residues in CDR region of VHH N1 are colored in red.

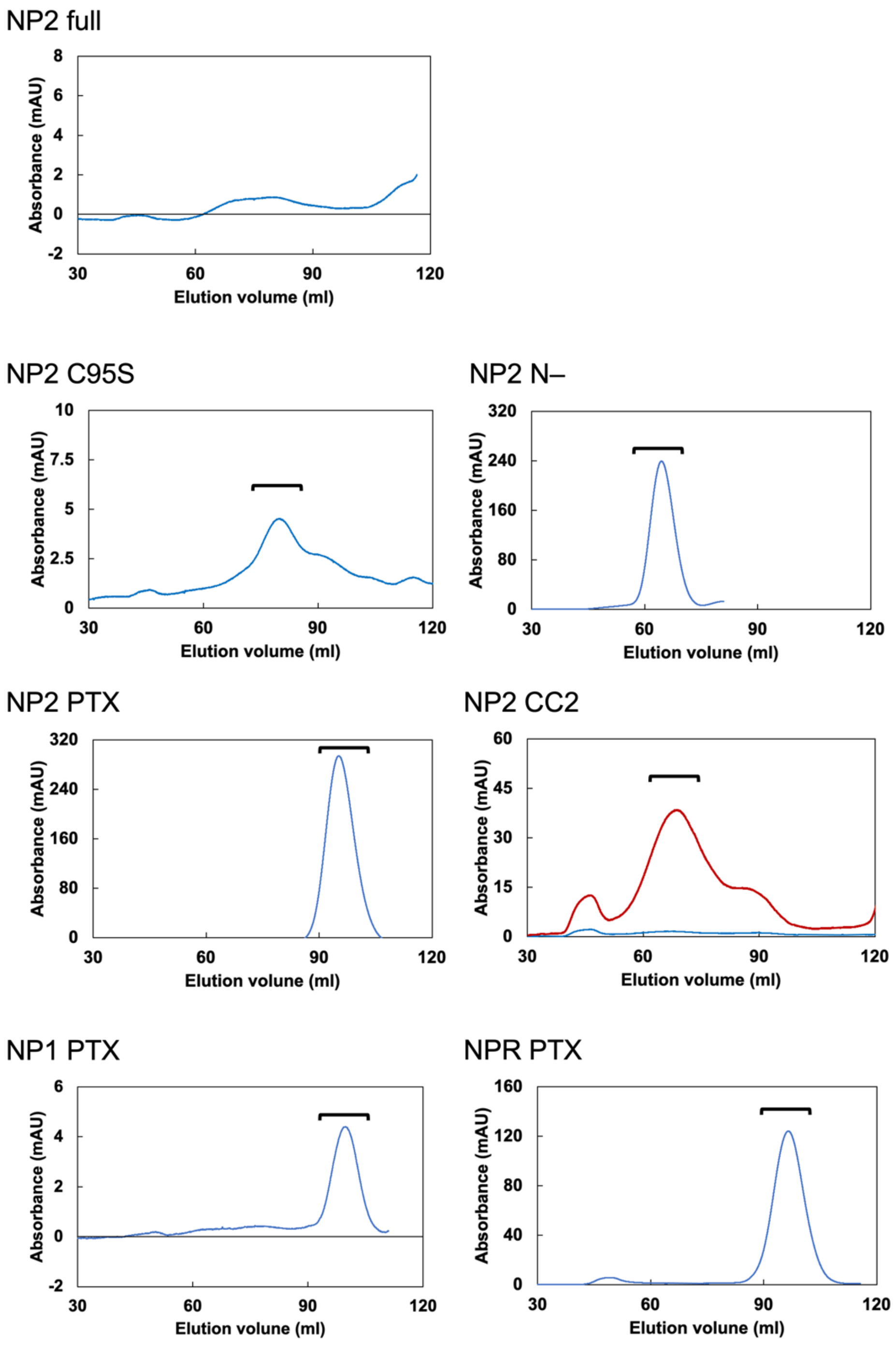

Figure. S2 Purification of NP constructs. Blue lines and red lines show the absorbance of 280 nm and 230 nm wavelength, respectively. Since NP2 CC2 does not contain any tyrosine or tryptophan residues and cannot be quantified by absorbance at 280 nm, absorbance of 230 nm derived from peptide bond was used for detection in SEC, and the final concentration was determined by the BCA method.

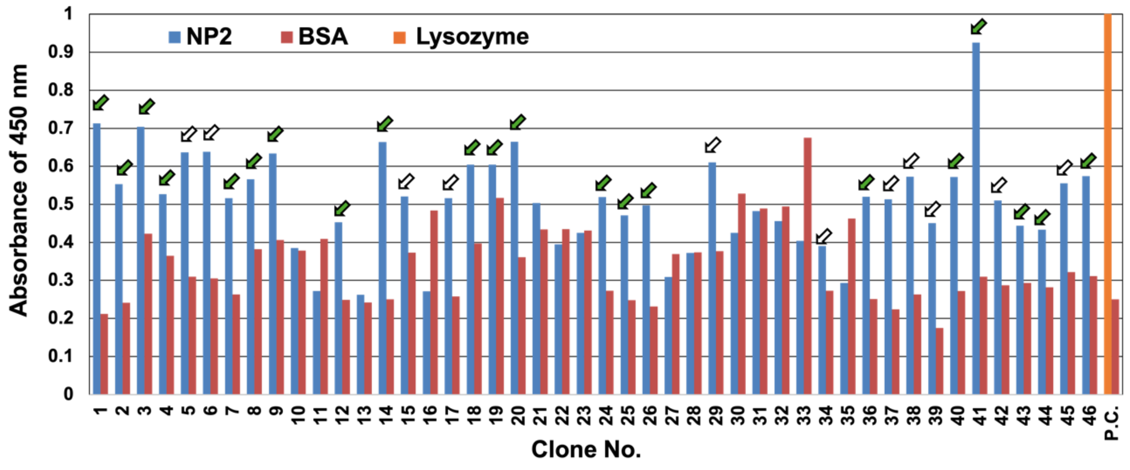

Figure S3. The result of the single clone phage ELISA. Lysozyme and VHH antibody against lysozyme was used as positive controls (P.C.). The blue, red, and orange bars represent the signals against NP2, BSA, and lysozyme for each clone, respectively. Clones whose sequences were analyzed are indicated by arrows, and clones whose sequence was same as VHH N1 were shown in green allow.

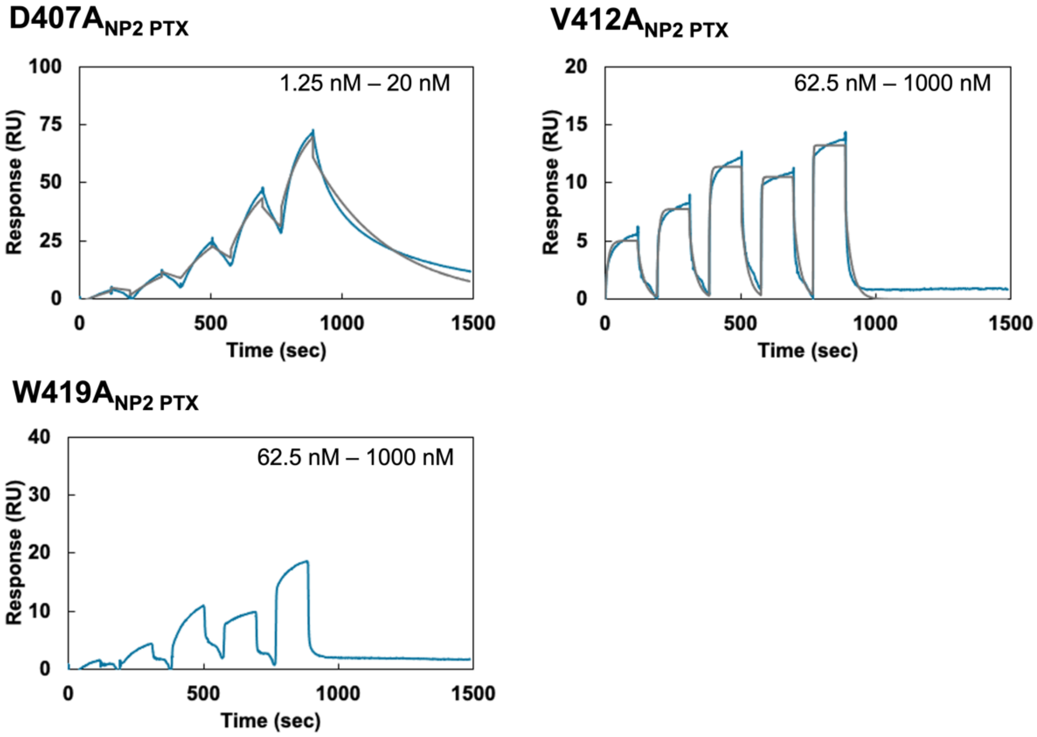

Figure S4. SPR sensorgrams of the interaction between VHH N1 and NP2 mutants. The measurements were conducted by single kinetic analyses. The blue line shows the raw data and the gray line shows the fitting data. All SPR measurements were performed in 10 mM HEPES-NaOH at pH 7.4, 150 mM NaCl, and Tween 20 (0.005 %) with or without 3 mM $CaCl_2$ at 25°C. NPs were immobilized on a CM5 sensor chip, and VHH N1 was flowed as the analyte. Each measurement was performed three times independently and each figure shows a sensorgrams for one of the three experiments.

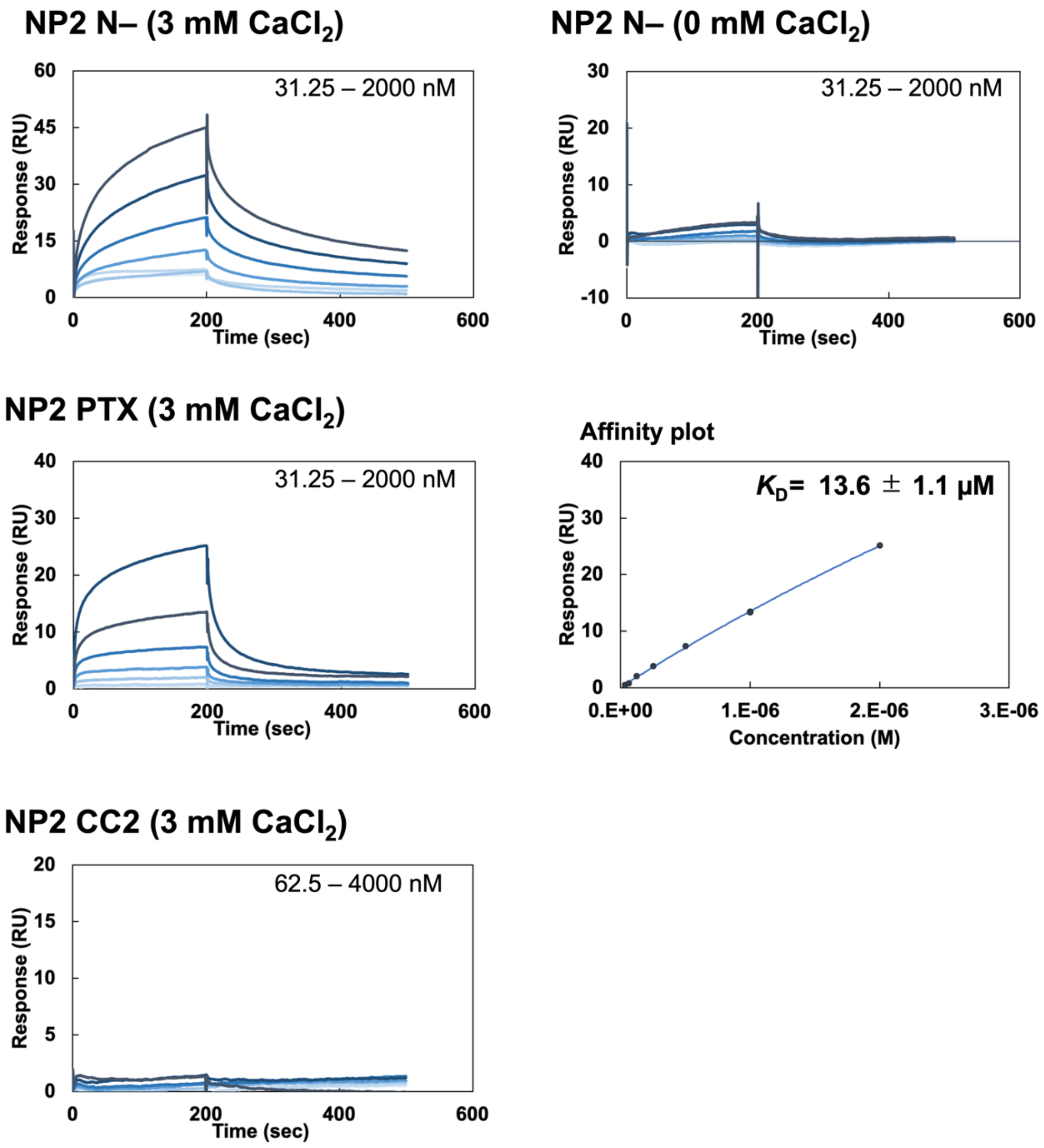

Figure S5. SPR sensorgrams of the interaction between GluA4 and NP2 constructs. The measurements were conducted by single kinetic analyses. The blue line shows the raw data and the gray line shows the fitting data. All SPR measurements were performed in 10 mM HEPES-NaOH at pH 7.4, 150 mM NaCl, and Tween 20 (0.005 %) with or without 3 mM $CaCl_2$ at 25°C. NPs were immobilized on a CM5 sensor chip, and VHH N1 was flowed as the analyte. Each measurement was performed three times independently and each figure shows a sensorgrams for one of the three experiments.

Table S1. Interface analyses of NB N1 using PDBePISA.

##	VHH N1	HSDC[a]	ASA[b]	BSA[c,d]	$\Delta^{i}G^{e}$
1	B:GLN 1		172.44	0.00	0.00
2	B:LEU 2		12.19	0.00	0.00
3	B:GLN 3		130.11	0.00	0.00
4	B:LEU 4		9.82	0.00	0.00
5	B:VAL 5		93.55	0.00	0.00
6	B:GLU 6		15.92	0.00	0.00
7	B:SER 7		49.17	0.00	0.00
8	B:GLY 8		37.36	0.00	0.00
9	B:GLY 9		29.64	0.00	0.00
10	B:GLY 10		25.30	0.00	0.00
11	B:LEU 11		163.74	0.00	0.00
12	B:VAL 12		27.67	0.00	0.00
13	B:GLN 13		80.59	0.00	0.00
14	B:ALA 14		35.77	0.00	0.00
15	B:GLY 15		54.74	0.00	0.00
16	B:ASP 16		65.62	0.00	0.00
17	B:SER 17		59.44	0.00	0.00
18	B:LEU 18		37.24	0.00	0.00
19	B:LYS 19		102.14	0.00	0.00
20	B:LEU 20		0.00	0.00	0.00
21	B:SER 21		21.03	0.00	0.00
22	B:CYS 22		0.00	0.00	0.00
23	B:ILE 23		81.90	0.00	0.00
24	B:ASP 24		3.37	0.00	0.00
25	B:SER 25		94.85	0.00	0.00
26	B:GLY 26		35.63	0.00	0.00
27	B:SER 27		99.54	0.00	0.00
28	B:THR 28		54.04	0.00	0.00
29	B:PHE 29		2.21	0.00	0.00
30	B:ASN 30		79.36	0.00	0.00
31	B:LEU 31		89.04	0.00	0.00
32	B:TYR 32		12.35	0.00	0.00
33	B:ALA 33		38.58	21.98 ||||||	0.35
34	B:MET 34		2.34	2.34 |||||||||||	0.04
35	B:GLY 35		4.45	4.45 |||||||||||	0.06
36	B:TRP 36		0.00	0.00	0.00
37	B:PHE 37		35.18	23.41 |||||||	0.37
38	B:ARG 38		20.20	0.00	0.00
39	B:GLN 39		64.44	0.00	0.00
40	B:ALA 40		22.99	0.00	0.00

<table>
<tr><td>41</td><td>B:PRO 41</td><td></td><td>109.84</td><td>0.00</td><td>0.00</td></tr>
<tr><td>42</td><td>B:GLY 42</td><td></td><td>90.24</td><td>0.00</td><td>0.00</td></tr>
<tr><td>43</td><td>B:LYS 43</td><td></td><td>116.78</td><td>0.00</td><td>0.00</td></tr>
<tr><td>44</td><td>B:GLU 44</td><td></td><td>147.61</td><td>60.34 |||||</td><td>0.41</td></tr>
<tr><td>45</td><td>B:ARG 45</td><td></td><td>97.11</td><td>18.17 ||</td><td>-0.21</td></tr>
<tr><td>46</td><td>B:GLU 46</td><td></td><td>68.24</td><td>23.67 ||||</td><td>0.25</td></tr>
<tr><td>47</td><td>B:PHE 47</td><td></td><td>65.23</td><td>61.08 ||||||||||</td><td>0.88</td></tr>
<tr><td>48</td><td>B:VAL 48</td><td></td><td>0.00</td><td>0.00</td><td>0.00</td></tr>
<tr><td>49</td><td>B:ALA 49</td><td></td><td>0.00</td><td>0.00</td><td>0.00</td></tr>
<tr><td>50</td><td>B:SER 50</td><td>H</td><td>25.42</td><td>25.42 |||||||||||</td><td>0.19</td></tr>
<tr><td>51</td><td>B:ILE 51</td><td></td><td>7.75</td><td>1.57 |||</td><td>0.03</td></tr>
<tr><td>52</td><td>B:SER 52</td><td></td><td>37.21</td><td>16.53 |||||</td><td>0.26</td></tr>
<tr><td>53</td><td>B:ARG 53</td><td></td><td>128.22</td><td>0.00</td><td>0.00</td></tr>
<tr><td>54</td><td>B:GLY 54</td><td></td><td>66.31</td><td>0.00</td><td>0.00</td></tr>
<tr><td>55</td><td>B:GLY 55</td><td></td><td>29.67</td><td>0.00</td><td>0.00</td></tr>
<tr><td>56</td><td>B:GLY 56</td><td></td><td>77.71</td><td>0.00</td><td>0.00</td></tr>
<tr><td>57</td><td>B:SER 57</td><td></td><td>44.76</td><td>0.33 |</td><td>0.01</td></tr>
<tr><td>58</td><td>B:SER 58</td><td></td><td>47.95</td><td>5.33 ||</td><td>-0.06</td></tr>
<tr><td>59</td><td>B:TYR 59</td><td></td><td>125.39</td><td>99.60 ||||||||</td><td>1.25</td></tr>
<tr><td>60</td><td>B:TYR 60</td><td>H</td><td>44.29</td><td>27.23 |||||||</td><td>-0.23</td></tr>
<tr><td>61</td><td>B:ALA 61</td><td>H</td><td>48.14</td><td>44.79 ||||||||||</td><td>-0.08</td></tr>
<tr><td>62</td><td>B:ASP 62</td><td></td><td>72.05</td><td>39.11 ||||||</td><td>0.33</td></tr>
<tr><td>63</td><td>B:SER 63</td><td>H</td><td>91.35</td><td>33.80 ||||</td><td>-0.05</td></tr>
<tr><td>64</td><td>B:GLN 64</td><td></td><td>71.90</td><td>0.00</td><td>0.00</td></tr>
<tr><td>65</td><td>B:LEU 65</td><td></td><td>5.01</td><td>0.00</td><td>0.00</td></tr>
<tr><td>66</td><td>B:LYS 66</td><td></td><td>123.70</td><td>22.45 ||</td><td>-0.33</td></tr>
<tr><td>67</td><td>B:GLY 67</td><td></td><td>64.70</td><td>0.00</td><td>0.00</td></tr>
<tr><td>68</td><td>B:ARG 68</td><td></td><td>32.33</td><td>0.00</td><td>0.00</td></tr>
<tr><td>69</td><td>B:PHE 69</td><td></td><td>1.59</td><td>0.00</td><td>0.00</td></tr>
<tr><td>70</td><td>B:THR 70</td><td></td><td>71.70</td><td>0.00</td><td>0.00</td></tr>
<tr><td>71</td><td>B:ILE 71</td><td></td><td>7.42</td><td>0.00</td><td>0.00</td></tr>
<tr><td>72</td><td>B:SER 72</td><td></td><td>41.06</td><td>0.00</td><td>0.00</td></tr>
<tr><td>73</td><td>B:ARG 73</td><td></td><td>28.48</td><td>0.00</td><td>0.00</td></tr>
<tr><td>74</td><td>B:ASP 74</td><td></td><td>52.19</td><td>0.00</td><td>0.00</td></tr>
<tr><td>75</td><td>B:ASN 75</td><td></td><td>49.66</td><td>0.00</td><td>0.00</td></tr>
<tr><td>76</td><td>B:ALA 76</td><td></td><td>101.48</td><td>0.00</td><td>0.00</td></tr>
<tr><td>77</td><td>B:LYS 77</td><td></td><td>134.96</td><td>0.00</td><td>0.00</td></tr>
<tr><td>78</td><td>B:ASN 78</td><td></td><td>26.90</td><td>0.00</td><td>0.00</td></tr>
<tr><td>79</td><td>B:THR 79</td><td></td><td>6.25</td><td>0.00</td><td>0.00</td></tr>
<tr><td>80</td><td>B:VAL 80</td><td></td><td>0.00</td><td>0.00</td><td>0.00</td></tr>
<tr><td>81</td><td>B:TYR 81</td><td></td><td>43.00</td><td>0.00</td><td>0.00</td></tr>
<tr><td>82</td><td>B:LEU 82</td><td></td><td>0.00</td><td>0.00</td><td>0.00</td></tr>
<tr><td>83</td><td>B:GLN 83</td><td></td><td>52.99</td><td>0.00</td><td>0.00</td></tr>
<tr><td>84</td><td>B:MET 84</td><td></td><td>0.00</td><td>0.00</td><td>0.00</td></tr>
<tr><td>85</td><td>B:ASN 85</td><td></td><td>58.25</td><td>0.00</td><td>0.00</td></tr>
<tr><td>86</td><td>B:SER 86</td><td></td><td>59.70</td><td>0.00</td><td>0.00</td></tr>
</table>

87	B:LEU 87		1.84	0.00	0.00
88	B:LYS 88		70.85	0.00	0.00
89	B:PRO 89		63.80	0.00	0.00
90	B:GLU 90		112.12	0.00	0.00
91	B:ASP 91		0.25	0.00	0.00
92	B:THR 92		28.34	0.00	0.00
93	B:ALA 93		1.16	0.00	0.00
94	B:VAL 94		41.36	0.00	0.00
95	B:TYR 95		0.00	0.00	0.00
96	B:TYR 96		35.92	0.00	0.00
97	B:CYS 97		0.00	0.00	0.00
98	B:ASN 98		3.09	3.09 ||||||||||	-0.04
99	B:ALA 99		0.00	0.00	0.00
100	B:ARG 100		116.67	59.90 ||||||	-0.99
101	B:ALA 101		28.34	0.00	0.00
102	B:GLY 102		83.29	0.00	0.00
103	B:ARG 103		234.98	4.73		-0.08
104	B:TYR 104		147.41	0.00	0.00
105	B:ASP 105		64.58	0.00	0.00
106	B:TYR 106		41.36	0.00	0.00
107	B:TRP 107		82.14	0.00	0.00
108	B:GLY 108		6.61	0.00	0.00
109	B:GLN 109		164.79	0.00	0.00
110	B:GLY 110		20.09	0.00	0.00
111	B:THR 111		18.09	0.00	0.00
112	B:GLN 112		84.73	0.00	0.00
113	B:VAL 113		0.00	0.00	0.00
114	B:THR 114		45.08	0.00	0.00
115	B:VAL 115		5.28	0.00	0.00
116	B:SER 116		31.92	0.00	0.00
117	B:SER 117		72.77	0.00	0.00
118	B:ALA 118		70.78	0.00	0.00
119	B:ALA 119		70.09	0.00	0.00
120	B:GLY 120		84.48	0.00	0.00
121	B:HIS 121		163.72	0.00	0.00

a. Residues making Hydrogen bond, Disulfide bond, Salt bridge or Covalent link
b. Solvent Accessible Surface Area
c. Buried Surface Area
d. Buried Surface percentage, one bar per 10 %
e. Solvent energy effect

Table S2. Interface analyses of NP2 PTX using PDBePISA.

##	NP2 PTX	HSDC[a]	ASA[b]	BSA[c,d]	Δ^iG^e
1	C:PHE 4		54.85	27.05 |||||	0.34
2	C:LYS 5		24.09	0.00	0.00
3	C:VAL 6		0.00	0.00	0.00
4	C:SER 7		19.89	10.82 ||||||	-0.06
5	C:LEU 8		0.33	0.00	0.00
6	C:PRO 9		86.88	1.84 |	0.03
7	C:LEU 10		101.82	0.00	0.00
8	C:ARG 11		85.43	0.00	0.00
9	C:THR 12		6.50	0.00	0.00
10	C:ASN 13		85.26	0.00	0.00
11	C:TYR 14		115.17	39.79 ||||	0.39
12	C:LEU 15		7.56	0.00	0.00
13	C:TYR 16		38.80	0.00	0.00
14	C:GLY 17		0.00	0.00	0.00
15	C:LYS 18		61.17	0.00	0.00
16	C:ILE 19		5.82	0.00	0.00
17	C:LYS 20	H	134.81	28.09 |||	-1.04
18	C:LYS 21		96.31	0.00	0.00
19	C:THR 22		64.83	0.00	0.00
20	C:LEU 23		2.05	0.00	0.00
21	C:PRO 24		57.70	0.00	0.00
22	C:GLU 25		87.84	0.00	0.00
23	C:LEU 26		4.42	0.00	0.00
24	C:TYR 27		124.27	0.00	0.00
25	C:ALA 28		18.81	0.00	0.00
26	C:PHE 29		0.31	0.00	0.00
27	C:THR 30		0.00	0.00	0.00
28	C:ILE 31		1.26	0.00	0.00
29	C:CYS 32		0.17	0.00	0.00
30	C:LEU 33		0.50	0.00	0.00
31	C:TRP 34		5.38	0.00	0.00
32	C:LEU 35		0.00	0.00	0.00
33	C:ARG 36		81.18	4.89 |	-0.18
34	C:SER 37		0.68	0.00	0.00
35	C:SER 38		81.18	0.00	0.00
36	C:ALA 39		15.51	0.00	0.00
37	C:SER 40		86.12	0.00	0.00
38	C:PRO 41		86.17	0.00	0.00
39	C:GLY 42		5.12	0.00	0.00
40	C:ILE 43		0.00	0.00	0.00
41	C:GLY 44		0.81	0.00	0.00
42	C:THR 45		0.00	0.00	0.00
43	C:PRO 46		0.24	0.00	0.00

44	C:PHE 47		0.15	0.00	0.00
45	C:SER 48		0.00	0.00	0.00
46	C:TYR 49		0.00	0.00	0.00
47	C:ALA 50		0.00	0.00	0.00
48	C:VAL 51		3.55	0.00	0.00
49	C:PRO 52		106.53	0.00	0.00
50	C:GLY 53		85.82	0.00	0.00
51	C:GLN 54		38.03	0.00	0.00
52	C:ALA 55		20.59	0.00	0.00
53	C:ASN 56		31.58	0.00	0.00
54	C:GLU 57		0.00	0.00	0.00
55	C:ILE 58		1.18	0.00	0.00
56	C:VAL 59		0.34	0.00	0.00
57	C:LEU 60		0.00	0.00	0.00
58	C:ILE 61		13.61	0.00	0.00
59	C:GLU 62		22.85	0.00	0.00
60	C:TRP 63		120.96	0.00	0.00
61	C:GLY 64		26.11	0.00	0.00
62	C:ASN 65		140.69	0.00	0.00
63	C:ASN 66		76.04	0.00	0.00
64	C:PRO 67		77.17	0.00	0.00
65	C:ILE 68		5.08	0.00	0.00
66	C:GLU 69		53.38	0.00	0.00
67	C:LEU 70		0.33	0.00	0.00
68	C:LEU 71		19.71	0.00	0.00
69	C:ILE 72		0.00	0.00	0.00
70	C:ASN 73		35.13	0.00	0.00
71	C:ASP 74		77.99	0.00	0.00
72	C:LYS 75		106.55	0.00	0.00
73	C:VAL 76		85.02	0.00	0.00
74	C:ALA 77		15.74	0.00	0.00
75	C:GLN 78		113.67	0.00	0.00
76	C:LEU 79		12.49	0.00	0.00
77	C:PRO 80		33.75	0.00	0.00
78	C:LEU 81		7.15	0.00	0.00
79	C:PHE 82		145.27	0.00	0.00
80	C:VAL 83		2.10	0.00	0.00
81	C:SER 84		44.84	0.00	0.00
82	C:ASP 85		75.16	0.00	0.00
83	C:GLY 86		34.42	0.00	0.00
84	C:LYS 87		126.96	0.00	0.00
85	C:TRP 88		4.30	0.00	0.00
86	C:HIS 89		20.93	0.00	0.00
87	C:HIS 90		1.46	0.00	0.00
88	C:ILE 91		0.16	0.00	0.00
89	C:CYS 92		0.00	0.00	0.00

90	C:ILE 93		0.33	0.00	0.00
91	C:THR 94		1.48	0.00	0.00
92	C:TRP 95		0.17	0.00	0.00
93	C:THR 96		26.48	0.00	0.00
94	C:THR 97		12.78	0.00	0.00
95	C:ARG 98		170.86	0.00	0.00
96	C:ASP 99		73.39	0.00	0.00
97	C:GLY 100		0.00	0.00	0.00
98	C:MET 101		91.15	0.00	0.00
99	C:TRP 102		19.72	0.00	0.00
100	C:GLU 103		41.54	0.00	0.00
101	C:ALA 104		0.00	0.00	0.00
102	C:PHE 105		5.63	0.00	0.00
103	C:GLN 106		22.03	0.00	0.00
104	C:ASP 107		59.69	0.00	0.00
105	C:GLY 108		11.54	0.00	0.00
106	C:GLU 109		110.50	0.00	0.00
107	C:LYS 110		109.13	0.00	0.00
108	C:LEU 111		64.44	0.00	0.00
109	C:GLY 112		20.08	0.00	0.00
110	C:THR 113		77.83	0.00	0.00
111	C:GLY 114		18.69	0.00	0.00
112	C:GLU 115		122.13	0.00	0.00
113	C:ASN 116		110.83	0.00	0.00
114	C:LEU 117		4.01	0.00	0.00
115	C:ALA 118		6.26	0.00	0.00
116	C:PRO 119		41.89	0.00	0.00
117	C:TRP 120		159.37	0.00	0.00
118	C:HIS 121		28.39	0.00	0.00
119	C:PRO 122		42.26	0.00	0.00
120	C:ILE 123		0.31	0.00	0.00
121	C:LYS 124		77.37	0.00	0.00
122	C:PRO 125		68.39	0.00	0.00
123	C:GLY 126		51.08	0.00	0.00
124	C:GLY 127		3.56	0.00	0.00
125	C:VAL 128		34.15	0.00	0.00
126	C:LEU 129		0.65	0.00	0.00
127	C:ILE 130		0.00	0.00	0.00
128	C:LEU 131		0.34	0.00	0.00
129	C:GLY 132		0.49	0.00	0.00
130	C:GLN 133		1.46	0.00	0.00
131	C:GLU 134		7.34	0.00	0.00
132	C:GLN 135		0.00	0.00	0.00
133	C:ASP 136		65.20	0.00	0.00
134	C:THR 137		60.37	0.00	0.00
135	C:VAL 138		64.90	0.00	0.00

136	C:GLY 139		29.02	0.00	0.00
137	C:GLY 140		15.23	0.00	0.00
138	C:ARG 141		169.89	0.00	0.00
139	C:PHE 142		32.86	0.00	0.00
140	C:ASP 143		45.83	0.00	0.00
141	C:ALA 144		35.24	0.00	0.00
142	C:THR 145		84.76	0.00	0.00
143	C:GLN 146		16.73	0.00	0.00
144	C:ALA 147		1.01	0.00	0.00
145	C:PHE 148		1.47	0.00	0.00
146	C:VAL 149		15.97	0.00	0.00
147	C:GLY 150		9.10	0.00	0.00
148	C:GLU 151		25.07	0.00	0.00
149	C:LEU 152		0.34	0.00	0.00
150	C:SER 153		0.00	0.00	0.00
151	C:GLN 154		54.04	0.00	0.00
152	C:PHE 155		0.34	0.00	0.00
153	C:ASN 156		6.91	0.00	0.00
154	C:ILE 157		0.33	0.00	0.00
155	C:TRP 158		13.36	0.00	0.00
156	C:ASP 159		60.79	0.00	0.00
157	C:ARG 160		102.81	0.00	0.00
158	C:VAL 161		60.65	0.00	0.00
159	C:LEU 162		3.61	0.00	0.00
160	C:ARG 163		156.24	0.00	0.00
161	C:ALA 164		59.84	0.00	0.00
162	C:GLN 165		97.75	0.00	0.00
163	C:GLU 166		38.56	0.00	0.00
164	C:ILE 167		0.00	0.00	0.00
165	C:ILE 168		76.05	0.00	0.00
166	C:ASN 169		47.01	0.00	0.00
167	C:ILE 170		25.89	0.00	0.00
168	C:ALA 171		9.96	0.00	0.00
169	C:ASN 172		96.95	0.00	0.00
170	C:CYS 173		34.86	0.00	0.00
171	C:SER 174		72.40	0.00	0.00
172	C:THR 175		42.39	0.00	0.00
173	C:ASN 176		112.42	0.00	0.00
174	C:MET 177		138.40	0.00	0.00
175	C:PRO 178		68.31	0.00	0.00
176	C:GLY 179		4.30	0.00	0.00
177	C:ASN 180		89.26	0.00	0.00
178	C:ILE 181		19.66	0.00	0.00
179	C:ILE 182		0.17	0.00	0.00
180	C:PRO 183		29.19	0.00	0.00
181	C:TRP 184		13.87	0.00	0.00

<table>
<tr><td>182</td><td>C:VAL 185</td><td></td><td>64.68</td><td>0.00</td><td>0.00</td></tr>
<tr><td>183</td><td>C:ASP 186</td><td>H</td><td>89.76</td><td>62.10 |||||||</td><td>-0.41</td></tr>
<tr><td>184</td><td>C:ASN 187</td><td></td><td>117.22</td><td>17.06 ||</td><td>0.27</td></tr>
<tr><td>185</td><td>C:ASN 188</td><td></td><td>6.38</td><td>0.00</td><td>0.00</td></tr>
<tr><td>186</td><td>C:VAL 189</td><td></td><td>0.00</td><td>0.00</td><td>0.00</td></tr>
<tr><td>187</td><td>C:ASP 190</td><td></td><td>44.18</td><td>33.88 ||||||||</td><td>-0.08</td></tr>
<tr><td>188</td><td>C:VAL 191</td><td>H</td><td>41.28</td><td>37.90 ||||||||||</td><td>0.17</td></tr>
<tr><td>189</td><td>C:PHE 192</td><td></td><td>72.92</td><td>12.94 ||</td><td>0.21</td></tr>
<tr><td>190</td><td>C:GLY 193</td><td></td><td>51.94</td><td>32.32 |||||||</td><td>0.03</td></tr>
<tr><td>191</td><td>C:GLY 194</td><td></td><td>53.48</td><td>33.86 |||||||</td><td>0.15</td></tr>
<tr><td>192</td><td>C:ALA 195</td><td></td><td>10.06</td><td>9.82 ||||||||||</td><td>-0.11</td></tr>
<tr><td>193</td><td>C:SER 196</td><td></td><td>33.55</td><td>31.58 ||||||||||</td><td>0.38</td></tr>
<tr><td>194</td><td>C:LYS 197</td><td>H</td><td>59.73</td><td>59.46 ||||||||||</td><td>-0.08</td></tr>
<tr><td>195</td><td>C:TRP 198</td><td></td><td>142.42</td><td>127.11 |||||||||</td><td>1.86</td></tr>
<tr><td>196</td><td>C:PRO 199</td><td></td><td>113.94</td><td>72.72 |||||||</td><td>1.16</td></tr>
<tr><td>197</td><td>C:VAL 200</td><td></td><td>38.47</td><td>0.00</td><td>0.00</td></tr>
<tr><td>198</td><td>C:GLU 201</td><td></td><td>48.70</td><td>0.00</td><td>0.00</td></tr>
<tr><td>199</td><td>C:THR 202</td><td></td><td>77.57</td><td>0.00</td><td>0.00</td></tr>
<tr><td>200</td><td>C:CYS 203</td><td></td><td>25.37</td><td>0.00</td><td>0.00</td></tr>
<tr><td>201</td><td>C:GLU 204</td><td></td><td>133.91</td><td>0.00</td><td>0.00</td></tr>
<tr><td>202</td><td>C:GLU 205</td><td></td><td>99.29</td><td>0.00</td><td>0.00</td></tr>
<tr><td>203</td><td>C:ARG 206</td><td></td><td>26.42</td><td>0.00</td><td>0.00</td></tr>
<tr><td>204</td><td>C:LEU 207</td><td></td><td>99.16</td><td>0.00</td><td>0.00</td></tr>
<tr><td>205</td><td>C:LEU 208</td><td></td><td>171.90</td><td>0.00</td><td>0.00</td></tr>
</table>

a. Residues making Hydrogen bond, Disulfide bond, Salt bridge or Covalent link
b. Solvent Accessible Surface Area
c. Buried Surface Area
d. Buried Surface percentage, one bar per 10 %
e. Solvent energy effect

Table S3. kinetic parameters of the interaction between VHH N1 and NP2 mutants.

NP constructs	k_{on} (10^5/Ms)	k_{off} (10^{-2}/s)	K_D (nM)
$WT_{NP2\ PTX}$	19.2 ± 0.2	0.208 ± 0.018	1.14 ± 0.25
$D407A_{NP2\ PTX}$	9.49 ± 1.40	0.305 ± 0.038	3.45 ± 0.79
$V412A_{NP2\ PTX}$	7.57 ± 0.18	4.04 ± 0.01	52.4 ± 0.2
$W419A_{NP2\ PTX}$	N.D.	N.D.	N.D.
